# 3D Printing‐Based Hydrogel Dressings for Wound Healing

**DOI:** 10.1002/advs.202404580

**Published:** 2024-11-18

**Authors:** Xuan Zhou, Xunzhou Yu, Tingting You, Baohua Zhao, Lanlan Dong, Can Huang, Xiaoqing Zhou, Malcolm Xing, Wei Qian, Gaoxing Luo

**Affiliations:** ^1^ Institute of Burn Research Southwest Hospital State Key Laboratory of Trauma and Chemical Poisoning Third Military Medical University (Army Medical University) Chongqing 400038 China; ^2^ Chongqing Key Laboratory for Disease Proteomics Chongqing 400038 China; ^3^ Department of Mechanical Engineering University of Manitoba Winnipeg MB R3T 2N2 Canada

**Keywords:** 3D printing, bioinks, hydrogel dressings, multi‐function, wound healing

## Abstract

Skin wounds have become an important issue that affects human health and burdens global medical care. Hydrogel materials similar to the natural extracellular matrix (ECM) are one of the best candidates for ideal wound dressings and the most feasible choices for printing inks. Distinct from hydrogels made by traditional technologies, which lack bionic and mechanical properties, 3D printing can promptly and accurately create hydrogels with complex bioactive structures and the potential to promote tissue regeneration and wound healing. Herein, a comprehensive review of multi‐functional 3D printing‐based hydrogel dressings for wound healing is presented. The review first summarizes the 3D printing techniques for wound hydrogel dressings, including photo‐curing, extrusion, inkjet, and laser‐assisted 3D printing. Then, the properties and design approaches of a series of bioinks composed of natural, synthetic, and composite polymers for 3D printing wound hydrogel dressings are described. Thereafter, the application of multi‐functional 3D printing‐based hydrogel dressings in a variety of wound environments is discussed in depth, including hemostasis, anti‐inflammation, antibacterial, skin appendage regeneration, intelligent monitoring, and machine learning‐assisted therapy. Finally, the challenges and prospects of 3D printing‐based hydrogel dressings for wound healing are presented.

## Graphical abstract

This review comprehensively describes the development of customizable multi‐functional 3D printing‐based hydrogel dressings in the field of wound healing, objectively evaluates the advantages and disadvantages from various perspectives, and proposes the core development concept, which is obviously distinct from previous work and will provide valuable information for the research and clinical transformation of 3D printing‐based hydrogel dressings for wound healing.

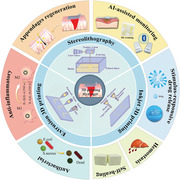

## 1 Introduction

The skin is the largest organ of the human body. It directly contacts the external environment and plays a barrier role in protecting the internal organs from external noxious stimuli, regulating physiological activities, and maintaining the balance of the body. However, damage to the skin's structural integrity and function caused by burn trauma, internal diseases, and iatrogenic injuries leads to the formation of wounds and poses a risk to human health.^[^ ^1^ ^]^ The healing process of a skin wound includes four main overlapping and interactive stages: hemostasis, inflammation, proliferation, and remodeling, during which any disorder may lead to the formation of chronic refractory wounds and excessive hypertrophic scars.^[^ ^2^ ^]^ Chronic wound patients are estimated to account for ≈2–6% of the global population, and their treatment accounts for ≈3–5.5% of global medical expenditures.^[^ ^3^ ^]^ The average annual cost of chronic wound treatment in the United States alone exceeds $20 billion.^[^ ^4^ ^]^ Therefore, promoting rapid and high‐quality wound healing is a vital development direction in skin tissue engineering.

Surgical and non‐surgical therapies are the two primary approaches to healing skin wounds. Surgical procedures include escharotomy, debridement, skin grafting, and skin flap transplantation, which are suitable for treating deep wounds. Non‐surgical treatments, including drug therapy, physiotherapy, and wound dressings, are appropriate for mild wounds or postoperative treatment, in which wound dressings can be regarded as temporary barriers that protect wounds from exogenous infections while providing a suitable microenvironment and coordinating wound tissue repair and regeneration.^[^ ^5^ ^]^ According to the theory of wet wound healing advanced by Winter in 1962,^[^ ^6^ ^]^ the ideal wound dressing should have good histocompatibility, moisture retention, physical and mechanical strength, and other comprehensive properties while maintaining certain biochemical properties to promote cell proliferation and migration to accelerate wound healing.^[^ ^7^ ^]^ Therefore, porous hydrogels similar to the natural extracellular matrix(ECM) are considered the most attractive dressings because of their adjustable physical, chemical, and biological properties, hydrophilicity, and good transparency.^[^ ^8^ ^]^ Hydrogels prepared by the traditional mold method have the disadvantages of fixed size, the lack of bionics, and poor mechanical properties.^[^ ^9^ ^]^ Although the injectable hydrogel developed subsequently improves the problem of adapting to the shape of the wound, the injection time is difficult to control. The uncertainty of the cross‐linking degree before and after injection may lead to problems such as syringe blockage or precursor diffusion, so the repeatability of injectable hydrogels needs to be further explored.^[^ ^10^ ^]^ In contrast, three‐dimensional (3D) printing technology can accurately create hydrogels that are customized to match any wound shape exactly according to the algorithm's design.^[^ ^11^ ^]^ 3D printing can also regulate the microstructure of printed hydrogel through layer‐by‐layer (LBL) augmentation manufacturing technology to obtain microporous structures that are highly similar to natural biological spatial tissue, which can accelerate cell proliferation and material transport, thus promoting tissue repair and regeneration.^[^ ^12^ ^]^ Compared with traditional manufacturing methods, 3D printing technology has greater flexibility, controllability, efficiency, and accuracy.

In recent years, 3D‐printing hydrogels have been widely studied by researchers in the field of wound healing.^[^ ^13^ ^]^ Some excellent reviews have discussed the progress of 3D printing hydrogels in the biomedical field. Wang et al.^[^ ^12a^ ^]^ introduced the application of a kind of 3D‐printing hydrogel based on extrusion technology for skin wound healing, especially for the vascularization and regeneration of skin attachments. Li et al.^[^ ^12b^ ^]^ highlighted the design and development of various biological inks and summarized the application of 3D‐printing hydrogels in regenerative medicine, in vitro disease modeling, surgical preparation, high‐throughput drug screening, and other biomedical fields. The current reviews mainly focus on 3D printing technology itself or discuss the specific function of 3D‐printing hydrogels and their multi‐directional applications in biology. However, with the integration of multiple disciplines, 3D printing‐based hydrogels are gradually developing toward integration and multi‐function. The application of multi‐functional 3D printing‐based hydrogel dressings for skin wound healing customized using 3D printing technologies has not been comprehensively summarized and analyzed.

**Figure 1:**
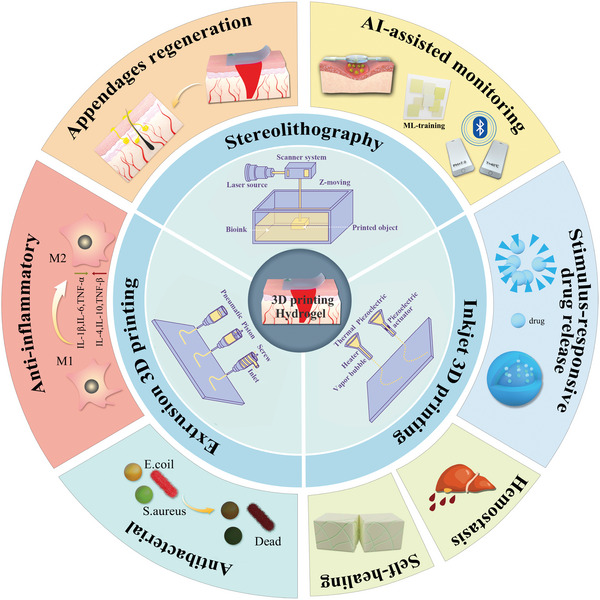
Overview of multifunctional 3D printing‐based hydrogel dressings customized by various 3D printing techniques.

This aims to be a comprehensive review focusing on multi‐functional 3D printing‐based hydrogel dressings for wound healing (**Figure** **1**). We introduce the process of wound healing, evaluation indexes, and mature 3D printing technology that can be used to manufacture hydrogel dressings. Representative 3D printing polymer materials for wound dressings are systematically classified and summarized. Most importantly, the latest progress on multi‐functional 3D printing‐based hydrogel dressings for wound healing is systematically expounded and objectively evaluated. Finally, the potential for future applications of 3D printing‐based hydrogel wound dressings is presented. This review comprehensively describes the development of customizable multi‐functional 3D printing‐based hydrogel dressings in the field of wound healing, objectively evaluates the advantages and disadvantages from various angles, and advances the core development concept, which is obviously distinct from previous work and will provide valuable information for the research and development of 3D printing‐based hydrogel dressings for wound healing.

## 2 Cutaneous Wound Healing

### 2.1 Skin Structure

As the largest organ of the human body, the skin not only maintains the balance and temperature regulation of the body but also protects the tissues and organs from the external environment.^[^ ^14^ ^]^ The epidermis is the visible outer layer of the skin in direct contact with the outside world. It is mainly composed of keratinocytes and has a barrier function.^[^ ^15^ ^]^ The epidermis is connected to the adjacent lower dermis through the basement membrane. The dermis is mainly divided into the papillary dermis and the reticular dermis and contains dense connective tissue, blood vessels, collagen, and fibers, providing the skin with mechanical strength and elasticity.^[^ ^16^ ^]^ Under the dermis is the subcutaneous tissue, which belongs to the innermost layer of the skin and is mainly composed of fat and connective tissue. Subcutaneous tissue secretes hormones, cushions mechanical pressure, and maintains constant body temperature.^[^ ^17^ ^]^

### 2.2 Wound Healing Process

**Figure 2:**
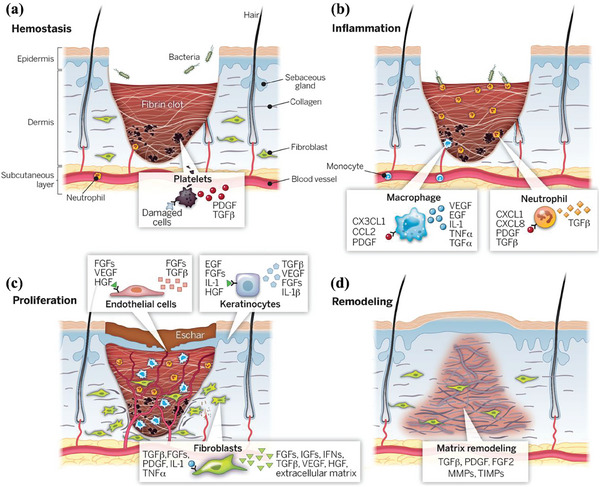
Skin structure and the four stages of wound healing: a) hemostasis, b) inflammation, c) proliferation, and d) remodeling. Reproduced with permission.^[^ ^17^ ^]^ Copyright 2014, American Association for the Advancement of Science.

Skin wound repair usually involves four dynamic and orderly processes: hemostasis, inflammation, proliferation, and remodeling.^[^ ^1^, ^13^, ^17^ ^]^ Once the skin is injured, platelets quickly adhere, accumulate at the wound site, and play a hemostatic role by forming fibrin (FIB) clots.^[^ ^18^ ^]^ The hemostatic period basically occurs at the same time as the inflammatory phase. During the inflammatory period, inflammatory cells, especially neutrophils and monocytes, are released to the wound site, remove foreign bodies, and secrete growth factors under phagocytosis, thus guiding the healing process into the proliferation stage.^[^ ^16^, ^17^ ^]^ At this time, the migration, proliferation, and differentiation of fibroblasts (FBs) and keratinocytes in the wound promote the synthesis of natural extracellular matrix (ECM), neovascularization, and granulation tissue.^[^ ^19^ ^]^ Then, the healing process reaches the final remodeling stage in which excess collagen fibers are degraded in the dermis, and the amount of type II collagen increases. Part of the mature granulation tissue further differentiates into myofibroblasts, which gather and contract at the edge of the wound over time to achieve the final repair of the skin wound.^[^ ^20^ ^]^ The skin structure and the four stages of wound healing are shown in **Figure** **2**.

### 2.3 Assessment of Wound Healing

Wound healing is a complex process. The effect of wound treatment should be evaluated objectively and accurately according to the corresponding indicators to allow for adjustments in the treatment plans in time to promote wound healing.

First, the wound healing speed is the most direct and macroscopic parameter with which to evaluate the effect of wound healing. The ratio of the wound healing area to the original wound area can be calculated. This value reflects the degree of wound healing and quantitatively evaluates the speed of wound healing.^[^ ^1^, ^21^ ^]^ Second, since the completion of re‐epithelialization is a particularly important wound healing index, the time of complete epithelialization (wound healing time) is also an indispensable traditional parameter for evaluating wound healing and can be intuitively observed by fixed‐point photography.^[^ ^22^ ^]^ The length of new epithelium, the thickness of granulation tissue, and the expression level of type I/III collagen can be measured by histological evaluation, hematoxylin‐eosin (HE) staining, and Masson staining to further evaluate the ability and degree of wound healing from the point of view of cellular biology.^[^ ^20a^ ^]^ Additionally, the regeneration of blood vessels and epidermal cells (EPCs) is critical in the proliferation and remodeling stage of wound healing. Therefore, the cellular content of proliferating cell nuclear antigen, e‐cadherin, or CD31 can be detected and analyzed using tissue immunochemistry, immunoblotting, and immunofluorescence. This content can reflect the proliferation, migration, and de‐adhesion of hair follicle EPCs, keratinocytes, and neovascularization cells and be used to assess the overall process of wound healing more accurately and professionally.^[^ ^23^ ^]^

## 3 3D Printing‐Based Hydrogel Dressings

### 3.1 Hydrogels versus Scaffolds for Wound Healing

The two typical structures of biomaterials that can be used for wound dressings are scaffolds and hydrogels.^[^ ^24^ ^]^ The choice of dressing structure depends on many factors, mainly the nature of the target tissue and the therapeutic drugs contained within. The main components of the present clinical, biological scaffolds for skin injury repair are collagen, such as Integra^[^ ^25^ ^]^ and Lando artificial dermis,^[^ ^26^ ^]^ which have made great achievements in the field of clinical wound repair. However, the mechanical properties of collagen‐based scaffolds are insufficient, and the degradation rate is too fast for the dynamic process of wound repair and, thus, cannot effectively induce tissue regeneration in situ. Therefore, researchers began to develop artificial scaffolds composed of artificial ECM, such as polycaprolactone (PCL), polylactic acid, and other hydrophobic materials, which are close to or simulate the structure and function of skin tissue.^[^ ^27^ ^]^ Artificial scaffolds with good mechanical properties can be implanted into tissue as load signaling factors and tissue regeneration carriers and provide mechanical support to maintain the stability of the entire structure.^[^ ^28^ ^]^ In contrast, hydrogels are hydrophilic polymers with a 3D network structure formed by the chemical or physical cross‐linking of natural or synthetic polymers, which are usually delivered to the wound by a syringe in a minimally invasive manner as a carrier for proteins, genes, or drugs.^[^ ^29^ ^]^ They can absorb a large amount of water while maintaining structural integrity, which is conducive to the absorption of wound exudates and the transport of oxygen and drugs, thus promoting the efficiency of wound healing.^[^ ^30^ ^]^ The unique advantage of hydrogel is that it provides a suitable microenvironment to promote cell survival and improve function.^[^ ^31^ ^]^ Therefore, biocompatible hydrogels with ideal properties, such as flexibility, water absorption, bio‐adhesion, and moisturizing functions, meet the basic requirements of skin ECM substitutes and are considered to be the most attractive tissue engineering materials.

Generally speaking, there are obvious differences between scaffolds and hydrogels in tissue engineering. First, in terms of chemical composition, scaffold materials are mostly composed of synthetic hydrophobic materials, such as PCL, polylactic acid, or dermis materials (Integra) from clinically available biological sources, whereas hydrogels are hydrophilic polymer networks composed of natural or synthetic polymers (polysaccharides, peptides, and glycosaminoglycans) that are cross‐linked chemically or physically.^[^ ^12^, ^32^ ^]^ Second, except for biological clinical dermal stents, artificial scaffolds generally have good mechanical properties, which provide mechanical support to maintain the stability of the tissue structure. Conversely, hydrogels exhibit relatively soft properties because of their porous network structure. More importantly, in terms of the mechanism of promoting wound repair, artificial scaffolds especially simulate the double‐layer structure of the skin epidermis and dermis and, thus, can be regarded as skin substitutes from the point of view of bionics,^[^ ^33^ ^]^ whereas hydrogels focus on their porous network structure as physical or biological factor carriers^[^ ^34^ ^]^ and tend to be used as skin wound dressings to provide a microenvironment suitable for wound cells and exert certain therapeutic and repair effects.

Although differences exist between scaffolds and hydrogels, their combination can form a composite structure that is beneficial for improving the overall properties of the materials.^[^ ^35^ ^]^ Stable hydrogel scaffolds with composite biological properties can be obtained by printing hydrogel material into the support scaffold using 3D printing, which not only provides channels for nutrient supply and material exchange in internal cells but also promotes tissue repair in situ through cell‐loaded hydrogels. Therefore, hydrogel scaffolds can be selectively combined with a variety of biomaterials to produce a biomimetic structure more suitable to the actual wound.

### 3.2 3D Printing Techniques for Hydrogels

Several traditional hydrogel manufacturing techniques are used in tissue engineering, including solvent casting, freeze‐drying, and electrospinning.^[^ ^36^ ^]^ Solvent casting is the simplest method to prepare hydrogel. It is based on the inversion technique of a mixed polymer solution, which has the problem of defective pore structures due to incomplete solute dissolution.^[^ ^36a^ ^]^ The freeze‐drying method is based on the principle of ice crystal sublimation to induce polymer phase separation and obtain porous hydrogels with high porosity. It has problems in that the size of the voids cannot be accurately adjusted, and the mechanical properties are generally poor^[^ ^36b^ ^]^ Electrospinning technology can be used to spray charged polymeric solution into an external high‐voltage electrostatic field to form fiber‐interconnected porous hydrogels. This method can control fiber diameter and porosity by adjusting the process parameters. However, the smaller pore size is not suitable for cell survival and biological behavior.^[^ ^36c^ ^]^ Therefore, tissue engineering hydrogels constructed by traditional methods generally have the disadvantages of fixed size, lack of bionics, and weak mechanical properties.

In recent years, 3D printing technology, which is another material manufacturing method that uses the aid of a 3D computer model, has been widely applied in the field of tissue engineering.^[^ ^37^ ^]^ The micro and macro structure of the material can be precisely customized using an algorithm, which makes it highly similar to natural ECM.^[^ ^38^ ^]^ The deposition of materials and cells can be controlled in space, which can help improve cell activity and promote tissue regeneration.^[^ ^28^, ^39^ ^]^ Therefore, biological 3D printing technology has the advantages of biological activity, flexibility, and high accuracy, which provides a new idea for manufacturing tissue‐engineered hydrogel. This review discusses four kinds of 3D printing technologies suitable for processing hydrogel dressing, including photo‐curing 3D printing based on a photopolymerization mechanism, extrusion 3D printing based on an extrusion strategy, inkjet 3D printing based on the printer principle, and laser‐assisted 3D printing based on laser energy deposition. **Table** **1** summarizes and tabulates the characteristics of these 3D printing techniques.

**Table 1:** A comparison of various 3D printing techniques.

3D printing techniques	Photo‐curing 3D printing			Extrusion 3D printing	Inkjet 3D printing	Laser‐assisted 3D printing
	SLA	DLP	TPP			
Description	Print fluid polymer ink layer by layer through the photon energy of UV/NIR irradiation			Bioinks are extruded from the mobile nozzle in the form of lines by pneumatic or mechanical driving force	Bioinks are deposited by a single droplet with a predetermined amount of ink driven by a thermal or piezoelectric device	Droplet bioinks excited by laser energy is transferred from the donor ribbon to the substrate
Accuracy (µm)	2–200	< 150	< 5	20—1000	2–100	≈10
Cell density	> 10^6^ cells mL^−1^	10^6^ to 10^8^ cells mL^−1^	Medium, 10^8^ cells mL^−1^	High, > 10^8^ cells mL^−1^	Low, < 10^6^ cells mL^−1^	Medium, 10^8^ cells mL^−1^
Cell viability	>75%	>75%	>85%	40–80%	≈90%	>95%
Ink viscosity	3–300 mPa s^−1^	1–200 mPa s^−1^	No limitation	30 mPa s^−1^‐60 kPa s^−1^	3–12 mPa/s	1–300 mPa s^−1^
Printing speed	Medium to fast	Fast	Fast	Slow	Fast	Medium
Cost	Low	High	High	Medium	Low	High
Advantages	High resolution, noncontact, wide range of biomaterial	High resolution, nozzle free, fast printing speed	High resolution, nozzle free, sub‐wavelength manufacture	High cell density, wide range of printable bioinks and versatility	Simple operation, fast printing speed, high resolution and low cost	High resolution, nozzle free, wide range of cell density and viscosity
Disadvantages	Incomplete transformation of reaction groups and biotoxicity	High cost, limited size and toxic photoinitiators	High cost, slow printing speed, limited biomaterial and biotoxicity	Limited printing accuracy, low printing speed, cell damage and low cell Viability	Limited viscosity range, low cell concentration and weak mechanical properties	High cost, toxicity, metallic particle contamination, complicated operation
Refs	[41, 42, 45, 57]	[42, 43, 57, 58]	[12, 44, 56]	[46, 48, 55, 59]	[38, 47, 50, 52, 60]	[38, 54, 57, 61]

#### 3.2.1 Photo‐Curing 3D Printing

**Figure 3:**
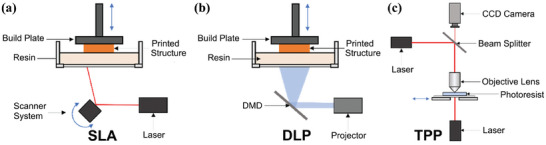
Schematic diagram of the photo‐curing 3D printing: a) SLA; b) DLP; c) TPP. Reproduced with permission.^[^ ^40^ ^]^ Copyright 2023, Elsevier.

Photo‐curing 3D printing is based on the growth and polymerization of the free radical chains of photosensitive materials under light irradiation^[^ ^40^ ^]^ and mainly includes three types: stereolithography (SLA), digital light processing (DLP), and two‐photon polymerization (TPP) (**Figure** **3**).

SLA is one of the earliest and most mature 3D printing technologies invented by Chuck Hull. SLA mainly uses the photon energy of ultraviolet (UV) light to print fluid polymeric ink layer by layer and produce 3D‐printed solid samples.^[^ ^41^ ^]^ The SLA system is mainly composed of a biological ink container, a UV light source to induce polymeric cross‐linking and a controllable mobile manufacturing platform.^[^ ^42^ ^]^ After the biological ink is loaded into the container, the focused UV beam is scanned and solidified point by point on the liquid surface according to the instructions programmed in the computer or scanner system. When the first layer of ink is irradiated and cured, the manufacturing platform drops one layer so that its surface is covered with a new layer of ink for the second layer of scanning. The former curing layer can be firmly polymerized with the newly cured layer through the unreacted functional groups on the surface. This process is repeated until the printing is completed, resulting in high‐resolution 3D‐printed samples.^[^ ^42a,c^ ^]^

Compared with SLA technology, DLP and TPP mainly improve the light source and imaging system, while the control and stepping system are similar. DLP is a variant of SLA created by Dr. Larry Hornback in 1987,^[^ ^43^ ^]^ which improves the printing accuracy and efficiency by introducing digital micromirror devices and transforming from a point light source to a surface light source.^[^ ^42b^ ^]^ TPP adopts the two‐photon absorption principle, which absorbs photons of two wavelengths (UV and near‐infrared (NIR)) at the same time through a suitable photoinitiator so that the non‐linear relationship between photon absorption energy and light intensity can be realized, thus avoiding the limitation of light diffraction, achieving sub‐wavelength manufacturing and further improving printing resolution.^[^ ^44^ ^]^ However, the photo‐curing 3D printing method still has some disadvantages, such as high cost, incomplete transformation of reaction groups, and certain biological toxicity of photoinitiators.^[^ ^41^, ^45^ ^]^

#### 3.2.2 Extrusion 3D Printing

**Figure 4:**
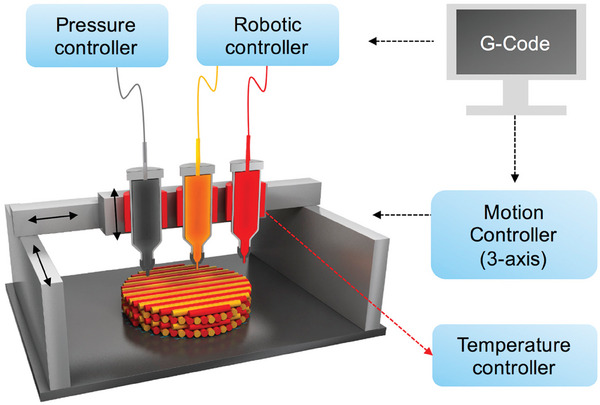
Schematic diagram of the extrusion 3D printing. Reproduced with permission.^[^ ^47a^ ^]^ Copyright 2016, Elsevier.

Extrusion 3D printing, also known as pressure‐assisted bio‐printing, is one of the widely used printing technologies in tissue engineering.^[^ ^46^ ^]^ Fluid polymeric bio‐ink is extruded from a syringe or mobile nozzle by a pneumatic or mechanical driving force and gradually deposited on the computer‐assisted design model platform as the print head moves along the X‐Y‐Z axis, as shown in **Figure** **4**. It is worth noting that biological inks need to maintain a certain viscosity and fluidity to maintain their structural shape before cross‐linking.^[^ ^47^ ^]^ In addition, polymeric biological inks for extrusion bioprinting can be pre‐mixed with cells, growth factors, or other active substances, so biological inks with high cell density (30 mPa s^−1^–60 kPa s^−1^) can be printed using this technology.^[^ ^48^ ^]^ Compared with other 3D printing processes, extrusion bioprinting provides higher cytocompatibility, flexibility, and versatility, but the resolution of the printed structure is relatively low. More importantly, the shear force and ink deformation at the nozzle are likely to cause cell damage or decrease cell activity.^[^ ^38^, ^49^ ^]^

#### 3.2.3 Inkjet 3D Printing

**Figure 5:**
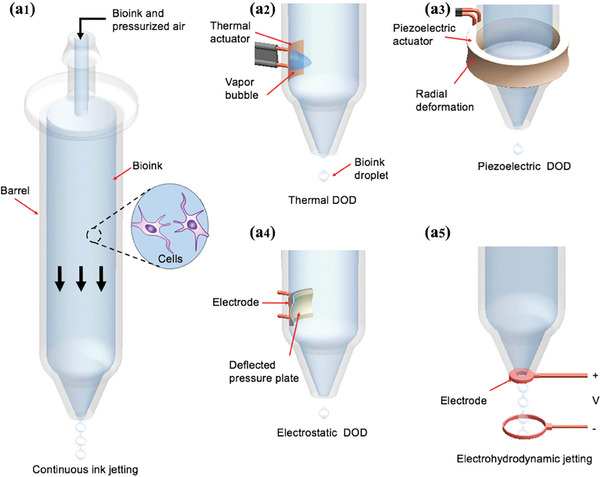
Schematic diagram of the inkjet 3D printing: (a1) continuous inkjet printing; (a2) thermal and (a3) piezoelectric drop‐on‐demand inkjet printing; (a4) electrostatic inkjet printing; (a5) electrohydrodynamic inkjet printing. Reproduced with permission.^[^ ^51b^ ^]^ Copyright 2016, Elsevier.

Inkjet 3D printing is a non‐contact material‐adding manufacturing technology developed from traditional industrial 2D printing and is one of the oldest approaches to biological printing.^[^ ^50^ ^]^ As shown in **Figure** **5**, inkjet 3D printing technology mainly includes continuous ink‐jet printing and on‐demand inkjet printing, among which drop‐on‐demand inkjet printing is more widely used. In the drop‐on‐demand titration inkjet printing system, biological ink is deposited by a single droplet with a predetermined amount of ink driven by a thermal or piezoelectric device.^[^ ^37^, ^51^ ^]^ Cells, growth factors, and other materials can be printed into high‐resolution structures alone or together using inkjet bio‐printing technology, which is suitable for the rapid manufacturing of scaffolds with complex structures.^[^ ^52^ ^]^ However, the range of ink viscosity that can be printed by inkjet printing technology is limited, and printing pressure may affect cell viability. This is coupled with the weak mechanical properties of the printing scaffold, which further hinders the development of this technology.^[^ ^47^, ^53^ ^]^

#### 3.2.4 Laser‐Assisted Printing (LAP)

**Figure 6:**
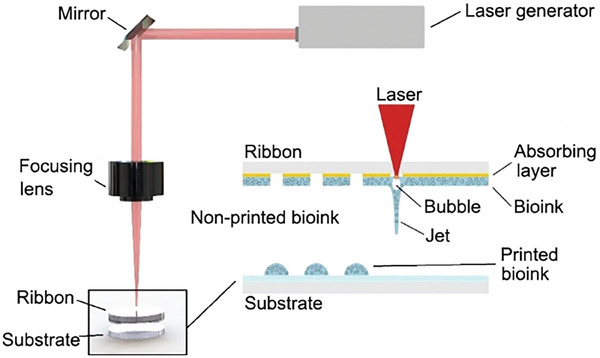
Schematic diagram of the LAP. Reproduced with permission.^[^ ^54^ ^]^ Copyright 2021, Wiley‐VCH.

LAP was originally used for precise metal deposition based on the principle of laser‐induced forward transfer and then successfully applied to deposit cells, DNA, and biomaterials in 2004.^[^ ^54^ ^]^ The LAP system mainly consists of a laser source, a ribbon coated with biological ink deposited on the metal absorption layer, and a receiving substrate (**Figure** **6**).^[^ ^12^, ^55^ ^]^ The metal absorption layer can convert laser irradiation to thermal energy. When the energy exceeds a certain threshold, the biological ink loaded with cells or factors evaporates and deposits on the receiving substrate in the form of droplets.^[^ ^12b^ ^]^ LAP is a nozzleless 3D printing technology that is compatible with a wide range of viscosities (1–300 mPa s^−1^) and effectively avoids the problem of biological ink clogging the sprinklers.^[^ ^56^ ^]^ In addition, compared with extrusion or inkjet printing, laser excitation has a great advantage in high‐resolution printing. However, many factors affect LAP resolution, including laser energy, wettability of the substrate, distance between the ribbon and substrate, viscosity, and the surface tension of ink droplets.^[^ ^55^ ^]^ Therefore, it is still necessary to regulate and control a variety of conditions accurately in practical applications. Furthermore, the metal absorption layer tends to break after absorbing a large amount of energy, and the resulting metal fragments are also at risk of contaminating the ink or damaging the cells, which is also a challenge that LAP technology needs to overcome.^[^ ^54^ ^]^

### 3.3 Bioinks for 3D Printing‐Based Hydrogel Dressings

Bioinks are special printing materials that contain biological components, such as living cells, ECM, and biological factors. The choice of bioink is essential for 3D printing‐based hydrogel dressings. The ideal bioink should possess several important characteristics, such as biocompatibility, appropriate rheological properties, and mechanical stability.^[^ ^32^ ^]^ The existing bioinks are mainly divided into natural polymers, synthetic polymers, and composite polymers. This section will summarize the advantages and disadvantages of these polymers and present their latest progress.

#### 3.3.1 Natural Polymer Ink

##### Collagen

Collagen is a natural protein that can be extracted from a variety of animal tissues. It is mainly distributed in the skin, cornea, muscle, blood vessels, and bone tissue.^[^ ^62^ ^]^ Because of its unique triple helix structure, collagen shows good mechanical properties.^[^ ^63^ ^]^ Similar to the biochemical and mechanical properties of natural ECM, collagen can participate in physiological and biochemical processes, such as cell proliferation, differentiation, migration, and signal transduction.^[^ ^64^ ^]^ Additionally, platelets can adhere to the surface of collagen to induce the release of coagulation factors and perform a hemostatic function.^[^ ^65^ ^]^ Therefore, collagen with appropriate mechanical properties, biological function, and biodegradability can be used as a 3D printing scaffold for cell attachment to directly regulate the wound microenvironment and promote the wound healing process.

##### Gelatin (Gel)

Gel is a natural peptide polymer obtained by the partial hydrolysis of collagen, which shows better biocompatibility and biodegradability than collagen, low immunogenicity, and no cytotoxicity.^[^ ^66^ ^]^ The structure of Gel is similar to that of the cell matrix and helps to promote cell adhesion, differentiation, proliferation, and migration.^[^ ^67^ ^]^ However, due to its reversible thermal response transition characteristics, Gel has poor thermal stability and is often used as a sacrificial material for 3D printing microchannel construction.^[^ ^68^ ^]^ Studies have shown that the mechanical strength of Gel can be improved by chemical or other modification. For example, methacrylic anhydride‐modified methacrylated gelatin (GelMA) obtained by in situ bioprinting has excellent cell growth ability and wound healing efficiency, showing great potential in 3D printing engineering.^[^ ^69^ ^]^

##### Silk

Silk is a protein material mainly derived from silkworms and spiders. It has good mechanical strength, biodegradability, and biocompatibility and contributes to cell adhesion and proliferation.^[^ ^70^ ^]^ Silk mainly contains natural silk fibroin (SF) and sericin, in which SF can be processed and developed into different forms of materials, such as fiber, film, sponge, foam, and hydrogel, and has wide application prospects in the field of tissue medicine.^[^ ^70^, ^71^ ^]^ However, the biological ink viscosity of SF alone is relatively low, so SF is mostly used as an additive or surface coating in combination with other polymers to prepare 3D printing‐based hydrogel dressings to improve the mechanical and biological properties of the materials in tissue construction.^[^ ^72^ ^]^ For example, SF/sodium alginate (SA) hydrogel were shown to have better mechanical properties and cell loading capacity than SA hydrogel and are suitable for 3D biological printing materials for wound healing.^[^ ^73^ ^]^

##### Hyaluronic Acid (HA)

HA is a kind of glycosaminoglycan composed of glucuronic acid and N‐acetyl‐D‐glucosamine alternately and is one of the main components of ECM.^[^ ^74^ ^]^ HA can be used as a component of biological ink to make an artificial skin structure with complete biocompatibility. HA exists widely in animal skin and tissues and can combine with a large number of water molecules to regulate the viscoelasticity of biological fluids.^[^ ^11^, ^75^ ^]^ HA can also participate in a variety of biological processes and play an important role in regulating cell behavior, reducing inflammation, as well as promoting angiogenesis and wound healing.^[^ ^76^ ^]^ However, HA degrades rapidly in vivo,^[^ ^77^ ^]^ so it is necessary to modify or covalently cross‐link the hydroxyl and carboxyl groups on the repeating units to improve its stability and expand its applications in tissue engineering.

##### Alginate (Alg)

Alg, derived from brown algae, is a kind of water‐soluble natural polysaccharide that is relatively non‐toxic, with low‐cost and good biocompatibility, so it is a suitable component of biological ink.^[^ ^78^ ^]^ Alg aqueous solutions can be induced by divalent cations (such as Ca^2+^, Mg^2+^, and Zn^2+^) to form physical hydrogels through cross‐linking, in which the gel rate, mechanical properties, and drug entrapment and release are controllable.^[^ ^79^ ^]^ In addition, Alg has the property of high shear thinning, which can flexibly change the material size.^[^ ^80^ ^]^ In contrast, because cells do not adhere to single Alg, cross‐linking with other cell adhesion groups is necessary to prepare hydrogel materials with good cell adhesion and mechanical properties.^[^ ^81^ ^]^ Therefore, in most cases, Alg needs to be prepared with other biopolymeric materials, such as polyethylene glycol (PEG) and polyethyleneimine, for 3D skin printing.

##### Chitosan (CS)

CS is a positively charged aminoglycan produced by the deacetylation of chitin in the hard shell of marine crustaceans. It is non‐toxic and has good biocompatibility, biodegradability, and antibacterial and hemostatic activity.^[^ ^82^ ^]^ As a wound dressing component, CS can ensure the wettability of the wound environment, reduce wound infections, and promote wound healing. These excellent properties make CS a good application prospect in skin tissue engineering.^[^ ^83^ ^]^ However, the low mechanical strength and low adhesion of CS limit its application in wound healing. Therefore, in in vivo and in vitro experiments, active groups, such as hydroxyl and amino groups on the main chain of CS, can be chemically modified or combined with other polymers using ions or chemical cross‐linking methods to improve the overall properties of CS materials.^[^ ^22^, ^84^ ^]^

##### Cellulose

Cellulose (C_6_H_10_O) is a kind of linear structure polysaccharide connected by a glycosidic bond and can be extracted from plants or bacteria as rich sources.^[^ ^85^ ^]^ Cellulose has good biocompatibility and biodegradability, which is suitable for use as a natural material in biomedical engineering.^[^ ^86^ ^]^ However, the disadvantage of insolubility in water limits the application of cellulose. Thus, cellulose must be solubilized by chemical modifications, such as carboxymethylation, silylation, and oxidation. Carboxymethyl cellulose (CMC), methylcellulose, hydroxypropyl cellulose, and carboxyethyl cellulose are all good choices for obtaining cellulose‐based hydrogels.^[^ ^87^ ^]^ Modified celluloses can also be regarded as bioink additives and combined with other polymers (such as Alg) to obtain 3D printing hydrogels with better properties.^[^ ^88^ ^]^ **Table** **2** summarizes the characteristics of natural bioinks for 3D printing‐based hydrogel dressings.

**Table 2:** Natural bioink for 3D printing‐based hydrogel dressings.

Natural bioink	Source	3D print techniques	Cross‐link mechanism	Advantages	Disadvantages	Application	References
Collagen	Animal tissue protein	Extrusion	Physical, hydrogen bond	Biocompatibility, cell affinity, Bioactive site	Rapid degradation, slow gelation rate, instability	Wound dressing, ECM scaffold	[64b]
Gelatin	Protein	Extrusion	Chemical, covalent bonds	Biocompatibility, non‐antigenic, cell affinity	Rapid degradation, Insufficient mechanical strength	Wound dressing, scaffold	[67c]
Silk	Silkworms and spiders	DLP	Chemical, photo‐initiated	Mechanical strength, biocompatibility, cell affinity	Low viscosity, complex extraction	Fiber scaffold, sponge, wound dressing	[71b]
Hyaluronic Acid	Animal tissue, Cockscombs, vitreous body	DLP	Chemical, photo‐initiated	Biocompatibility, water‐binding ability	Rapid degradation, Insufficient mechanical strength	Wound dressing, inflammation, angiogenesis	[11a]
Alginate	Natural seaweed	Inkjet	Physical, hydrogen bond, hydrophobic interaction	Non‐toxic, low‐cost, biocompatibility	Poor cell adhesion, inadequate support for cell proliferation	Wound dressing, patch, microneedle	[79c]
C hitosan	Deacetylation of chitin	SLA	Chemical, ionic	Biocompatibility, biodegradability, non‐toxicity	Insufficient mechanical strength and adhesion	Wound dressing, scaffold, anti‐bacteria, hemostasis	[84c]
Cellulose	Plants, natural fibers or bacteria	Extrusion	Chemical, thermal	Abundant source, biocompatibility, biodegradability	Poor flexibility and solubility, Low swelling rate	Wound dressing, patch, drug release	[87c]

The advantages of natural biomaterials originate from their excellent biological activities and help them adapt to the environment in the human body. However, natural polymers also have the disadvantages of weak mechanical properties and limited customization options. A variety of chemical strategies have been proposed to solve these problems, resulting in the development of synthetic polymers suitable for 3D printing‐based hydrogel dressings.

#### 3.3.2 Synthetic Polymer Ink

##### PEG

PEG is a biocompatible synthetic polymer with features of good fluidity, hydrophilicity, solubility, and adjustable cross‐linking degrees, which can regulate cell behavior. It is widely used in 3D printing in the form of hydrogel.^[^ ^89^ ^]^ Although PEG is not degradable and cannot be completely metabolized in the human body, polymeric chains with molecular weights of less than 30 kDa can be cleared through the kidneys.^[^ ^90^ ^]^ PEG has a molecular weight‐dependent melting point. For example, PEG400 is a colorless viscous liquid, whereas PEG2000 is a white solid with a melting point of ≈40 °C. The melting point of PEG increases with increases in molecular weight, which can be used to adjust the properties of 3D printing polymers at human body temperature.^[^ ^12b^ ^]^ In addition, PEG can be cross‐linked with other components, such as poly(ethylene glycol) diacrylate and poly(ethylene glycol) methacrylate hydrogels, to form copolymers adapted to various biomedical environments and have been used to develop biological patches.^[^ ^91^ ^]^

##### Polyvinyl Alcohol (PVA)

PVA is a widely used water‐soluble linear polymer with good biodegradability, biocompatibility, hydrophilicity, and high crystallinity. PVA can be cross‐linked by physical and chemical approaches to form hydrogel materials with good electrical conductivity, suggesting that it has promising application prospects in the fields of biomedicine and flexible sensing.^[^ ^92^ ^]^ In particular, PVA can be used to prepare hydrogels by freezing/thawing alternating cycle technology, which avoids the introduction of chemical cross‐linking agents, thus reducing the toxicity of hydrogels while improving elasticity and mechanical strength.^[^ ^93^ ^]^ In addition, PVA‐based hydrogels with good water retention capacity can maintain a moist environment during treatment and control the release of active substances, thus showing great potential in promoting skin wound healing.^[^ ^94^ ^]^

##### PCL

PCL is a biodegradable semicrystalline polyester with good biocompatibility, toughness, and easy processability, which makes it suitable for use in 3D‐printed tissue engineering scaffolds. PCL degrades slowly and can be used as a material for long‐term implants in vivo to induce tissue regeneration by providing support and material transport channels.^[^ ^95^ ^]^ PCL scaffolds also have good cytocompatibility and can further promote the proliferation and differentiation of cells in newborn tissues.^[^ ^95^, ^96^ ^]^ However, the hydrophobicity of PCL is disadvantageous to cell adhesion, so other organic polymers (such as nanocellulose and allylamine polymer) or biological activators, such as nerve growth factor and vascular endothelial growth factor (VEGF), can be introduced to enhance cell adhesion.^[^ ^97^ ^]^ Therefore, the degradation rate, antibacterial properties, and cell adhesion of PCL, which is conducive to accelerating the process of wound healing, must be considered. A concise summary of synthetic polymeric ink for 3D printing‐based hydrogel dressings is shown in **Table** **3**.

**Table 3:** Synthetic polymer ink for 3D printing‐based hydrogel dressings.

Synthetic polymer ink	Source	3D printing techniques	Cross‐link mechanism	Advantages	Disadvantages	Application	References
PEG	Petroleum product	DLP	Chemical, photo‐initiated	Fluidity, hydrophilicity, adjustable cross‐linking degree	Low cell affinity, incompletely degradable	Customized scaffold, wound dressing	[91a]
PVA	Water‐soluble linear polymer	Extrusion	Chemical, ionic, freeze‐thaw	Biocompatibility, biodegradability, hydrophilicity	Poor water resistance, insufficient mechanical strength, instability	Microneedle, sensor, wound dressing	[94a]
PCL	Caprolactone polymerization	Extrusion	Chemical, photo‐initiated	Biodegradable, biocompatibility, easy processability	Low cell affinity, long degradation time	Customized scaffold, wound dressing	[96]

#### 3.3.3 Composite Polymer Ink

Human tissue is essentially an anisotropic composite material composed of different components and structures. However, one‐component hydrogel 3D printing using a single natural bioink or synthetic polymeric ink has advantages and disadvantages and cannot fully meet the needs of biological applications. Specifically, natural polymers have high biocompatibility and biodegradability, which can promote cell adhesion, growth, and proliferation; however, their poor mechanical properties limit their development. In contrast, the microstructure, mechanical properties, and integrity of synthetic polymers can be artificially regulated, but the biocompatibility and biological activity of synthetic polymers are not as good as those of natural biomaterials.^[^ ^98^ ^]^ Therefore, the combination of natural biomaterials and synthetic polymers has been adopted to maximize their advantages and compensate for their respective shortcomings. In addition, the function of the composite hydrogel can be controlled by adjusting the proportion of the components to meet the needs of different organizational environments and has wide application prospects.

Compared with complex chemical modifications, the blending approach is a physical mixture of polymers with organic solvents. A composite matrix is then obtained by freeze‐drying or other techniques. This method can retain the function of each single component and has the advantages of simplicity, convenience, and low cost.^[^ ^99^ ^]^ The blending method includes three forms: natural‐natural polymer blending, natural‐synthetic polymer blending, and synthetic‐synthetic polymer blending. The researchers combined FIB with Gel to retain good cell adhesion and low immunogenicity while improving the mechanical strength of composite scaffolds.^[^ ^100^ ^]^ Collagen and sodium Alg were blended to form a unique cross‐linked interpenetrating polymeric network, which endowed the hybrid hydrogel with excellent biological activity and mechanical properties.^[^ ^101^ ^]^ A double‐network hydrogel was prepared by permeating polyaniline grafted with carboxymethyl CS and polyacrylamide (PAM) through multiple dynamic cross‐linking processes, which not only significantly increased the mechanical strength of the composite but also produced a multi‐functional system with electrical conductivity, adhesion, and antibacterial and biocompatibility properties^.[^ ^102^ ^]^ A new type of micromachining technology has been developed using the capillary force of SF. A PEG diacrylate/sucrose matrix was filled with SF scaffolds, and then high mechanical strength microneedles with controllable drug release were rapidly fabricated by photopolymerization.^[^ ^103^ ^]^ Generally speaking, various types of blending methods can be used to optimize the biological and mechanical properties of composite polymers, which have significant application value in biological tissue engineering and clinical fields.

### 3.4 Frontier Bioprinting Techniques and Biomaterials for Hydrogels

Hydrogels created by 3D printing technology can highly simulate tissue structures but lack the ability to accurately respond to dynamic environmental changes.^[^ ^57^, ^104^ ^]^ In recent years, in situ bioprinting that can accurately match the geometry of the wound surface and 4D bioprinting that includes the fourth dimension (time) have been gradually developed.^[^ ^32^, ^105^ ^]^ Along with the development of corresponding new biomaterials, some limitations of traditional 3D printing have been effectively compensated.

In situ bioprinting, also known as in vivo bioprinting, is a technology that directly deposits ink on tissue surfaces and cross‐links in situ.^[^ ^105^ ^]^ Hydrogels with appropriate structural stability and suitable shape to the wound shape can be obtained through this method, thereby reducing the cost of wound scanning and model creation.^[^ ^32^, ^106^ ^]^ There have been several reports of in situ bioprinting in various animal wound models.^[^ ^69^, ^107^ ^]^ Among them, handheld in situ bioprinting has the advantages of simplicity and convenience, but with low resolution and precision, while robot‐assisted in situ bioprinting can achieve automated manufacturing processes but is accompanied by high costs.^[^ ^108^ ^]^ As an emerging technology, in situ bioprinting has not yet been truly applied in clinical practice. On the one hand, how to maintain sterile conditions during the in‐situ printing process limits its application.^[^ ^105^ ^]^ In addition, the biomaterials required for in situ bioprinting contain a large quantity of cells, which requires in vitro cell expansion before surgery to obtain biomaterials with high concentrations of suitable cells.^[^ ^105^, ^106^ ^]^ Therefore, the ethical issues involved in this process need to be effectively solved in the future.

4D bioprinting can be considered the next generation additive manufacturing technology based on 3D printing, which allows the fabrication of 4D structures that can change shape according to external stimuli or over time.^[^ ^32^, ^57^ ^]^ As an emerging biomanufacturing technology, dynamic biological structures manufactured by high precision 4D bioprinting have unique capabilities such as self‐folding, twisting and unfolding, which can simulate the in vivo reactions of tissues more accurately.^[^ ^32^, ^109^ ^]^ The primary differences between 4D bioprinting and 3D printing relate to bioinks of stimulus‐responsive materials (also known as smart materials),^[^ ^110^ ^]^ which mainly include shape memory alloys and shape memory polymers that are physically responsive (such as temperature, humidity, light, electric fields or magnetic fields), chemically responsive (such as pH), and biologically responsive (such as glucose and enzymes).^[^ ^104^, ^111^ ^]^ In recent years, 4D bioprinting has attracted more and more attention in the printing of skin, blood vessels, bone and drug delivery media.^[^ ^112^ ^]^ However, the current selection of biocompatible smart biomaterials with good shape deformation capabilities required for 4D bioprinting is limited, which makes it difficult to respond to multiple stimuli at the same time and cannot meet the requirements of the complex human environment.^[^ ^57^, ^109^ ^]^ Therefore, 4D bioprinting is still in its infancy, and there is a long way to go before it can be truly applied in clinical practice.

## 4 Recent Progress in Multi‐Functional 3D Printing‐Based Hydrogel Dressings for Wound Healing

Due to the different appearances and manifestations of skin wounds, the clinical requirements for the function of wound dressings are complicated, such as promoting initial wound hemostasis as an adhesive and forming a barrier against inflammation and infection, delivering related therapeutic drugs, and the real‐time monitoring of wound healing. According to these clinical wound repair needs, various types of functionally modified hydrogels are rapidly developing. Therefore, this paper focuses on the recent progress of multi‐functional 3D printing‐based hydrogel dressings for wound healing.

### 4.1 Application Scene of 3D Printing‐Based Hydrogel Dressings

#### 4.1.1 Acute Wounds

Acute wounds mainly refer to sudden injuries, including physical injuries (such as abrasions, scratches, and friction contact injuries), iatrogenic injuries (such as surgical incisions and injection injuries), burns, electrical injuries, and chemical corrosion injuries.^[^ ^35^, ^113^ ^]^ Acute wounds refer to tissue trauma and can usually resolve in a short time. However, they are generally embedded with sundries that are difficult to clean, resulting in the risk of inflammation and bacterial infection.^[^ ^113^ ^]^ In addition, the wound forms an isolation layer after osmosis and scabbing, which cannot easily absorb drugs.^[^ ^19^, ^114^ ^]^ Therefore, for acute wounds, it is necessary to inhibit wound contraction and local inflammation while promoting fibrous tissue growth.

Yu et al.^[^ ^115^ ^]^ reported a rapid photo cross‐linking hydrogel wound dressing based on natural biomaterials. Similar to ECM in structure and composition, the dressing showed excellent cytocompatibility, anti‐oxidation, anti‐inflammatory, and antibacterial properties. In in vivo experiments, hydrogel dressings also showed enhanced angiogenesis and re‐epithelialization, which is beneficial to promoting the healing of acute skin wounds and suggests promising applications in acute wound dressings. Additionally, a bio‐mimicking hydrogel designed by Cai et al.^[^ ^116^ ^]^ used a thiol‐ene click reaction to capture the early physical signals triggered by inflammation and release chemokines, promoting the early polarization of macrophages in a rat model of acute trauma. Natural adhesion sites are provided as well to promote collagen deposition and angiogenesis, thus realizing the precise remodeling of soft tissue. Xu et al.^[^ ^117^ ^]^ prepared 3D bio‐printed hydrogel patches cross‐linked with GelMA and silk fibroin to improve the mechanical properties of hydrogels, which had good mechanical properties and biodegradability. In the treatment of acute wounds, hydrogel dressings promote the adhesion, proliferation, and migration of FBs while improving the microenvironment needed for tissue regeneration and collagen production to accelerate wound healing, providing an inexpensive and effective way to promote acute wound healing.

#### 4.1.2 Chronic Wounds

Chronic wounds refer to wounds that have not fully healed or recurred for a long time after trauma, including vascular ulcers, pressure sores, diabetic foot ulcers (DFU), and autoimmune system diseases. These wounds bring a great burden to patients and healthcare systems.^[^ ^3^, ^118^ ^]^ Chronic wounds show problems in many aspects. First, the surgical debridement of decaying tissue that is difficult to clean will lead to an increase in new wounds. Second, the isolation layer produced by a large amount of pus and scabbing reduces the effective absorption of drugs.^[^ ^118a^ ^]^ Bacterial infection is the main cause of slow wound healing. However, in turn, decaying tissue in chronic wounds contributes to bacterial growth.^[^ ^119^ ^]^ Finally, the accumulation of reactive oxygen species (ROS) in chronic wounds seriously damages the cellular structure, while oxidative stress prolongs inflammation, which is not conducive to wound healing.^[^ ^118^, ^119^ ^]^ Therefore, hydrogel dressings for chronic wounds generally have good antibacterial, antioxidant, anti‐inflammatory, and regenerative properties.

Guo et al.^[^ ^64b^ ^]^ developed a 3D‐printed natural hydrogel by physically cross‐linking the rich proteins in fresh egg whites. As a non‐cytotoxic dressing, it not only stimulated the proliferation and migration of FBs and stem cells but also promoted angiogenesis and collagen deposition, thus promoting the recovery of diabetic chronic wounds. Wu et al.^[^ ^120^ ^]^ reported a PAM/hydroxypropyl methylcellulose 3D‐printed hydrogel dressing loaded with silver nanoparticles (NPs). The unique hyper‐porous structure was conducive to the controlled release of silver NPs, which ensured biocompatibility and improved the antibacterial activity of the hydrogel dressing. Inspired by marine sponges, Kim et al.^[^ ^121^ ^]^ integrated salmon sperm DNA and DNA‐induced biological silica into hydrogel dressings using 3D printing to effectively absorb wound exudates. The nano‐therapeutic drugs in composite dressings have attractive application prospects in scavenging ROS, promoting angiogenesis, and improving anti‐inflammatory activity. Qiu et al.^[^ ^122^ ^]^ conducted related research on the prevention and treatment of pressure ulcers (PU). A nanofiber yarn (NFY) network composed of polyacrylonitrile (PAN) and reduced graphene oxide (rGO) was encapsulated into injectable 3D hydrogel scaffolds, which showed excellent cytocompatibility, self‐healing, and broad‐spectrum antibacterial activity. The electrical conductivity of hydrogel scaffolds can be used in wound pressure monitoring sensors to regulate cell tissue and accelerate wound healing.

### 4.2 Advanced Features of 3D Printing‐Based Hydrogel Dressings for Wound Healing

In this section, the recent advancements in multi‐functional 3D printing‐based hydrogel dressings customized by representative 3D printing technology are comprehensively reviewed. The related content is summarized and supplemented in **Table** **4**.

**Table 4:** Customizable multi‐functional 3D printing‐based hydrogel dressings.

Multi‐function	Ink materials	3D printing techniques	3D printing devices	Functional compounds	References
Hemostasis	Oxidized starch	Extrusion	Hydrogel	Coagulation factor	[13a]
	alkyl‐CS/SA/PEG	Extrusion	Microfibers	alkyl‐CS	[124b]
	CS/PLA	–	Sponge	CS	[126]
	GelMA	SLA	Sponge	Taper GelMA	[127]
	TCNF/CS	Extrusion	Scaffold	CS/casein	[128]
Anti‐inflammatory	ALG‐XAN	Extrusion	Hydrogel	Clove essential oil	[13b]
	SA/polymerized PAM	Inkjet	Hydrogel‐ scaffold	MOF nanozyme	[60]
	CMCs‐O‐AlgCat	Extrusion	Scaffold	Catechol group	[132]
	PLLA/CS	Extrusion	Scaffold	CS/PDA	[133]
	Methacrylated decellularized extracellular matrix	Extrusion	Scaffold	Copper‐epigallocatechin gallate	[163]
Antibacterial	MH‐Gel	Extrusion	Hydrogel	MH	[135]
	GelMA/CMC/XAN	Direct ink writing	Hydrogel	GCX‐CeO_2_	[138]
	CS/methacrylate	Extrusion	Hydrogel	Levofloxacin	[137]
	Dextran/MoS_2_	Extrusion	Hydrogel‐ scaffold	MoS_2_	[139]
	Alg‐DA	Co‐axial 3D printing	Hydrogel‐ scaffold	Copper ions	[164]
	TCNF	Extrusion	Scaffold	Epsilon‐poly‐L‐lysine	[165]
Self‐healing	Concentrated CMC	Extrusion	Scaffold	CMC	[87c]
	PVA/AAc/PEGDA	DLP	Hydrogel	PVA/AAc	[142]
	HAMA‐ PBA/GelMA	Extrusion	Hydrogel	HA‐DA	[143]
	HNT/PDA/PVA	Extrusion	Hydrogel	HNT/PDA	[144]
	SF/Oxidized salep/kappa carrageenan NPs	Extrusion	Hydrogel	SF/OS	[166]
Stimulus‐responsive drug release	PLA/LA/Mel	Extrusion	Scaffold	Mel	[33]
	PCLMA/HAMA/heparin‐SH	DLP	MN	HAMA/heparin‐SH	[58]
	Alg‐Gel/PCL/PDA	Extrusion	Hydrogel‐ scaffold	PDA	[147]
	Spidroin/PFKU/Aloe vera gel (avGel)	Extrusion	MN	Mxene	[148]
	Poly (n‐isopropylacrylamide)/PVA	Extrusion	Hydrogel	MoS_2_/Fe_3_O_4_	[167]
Appendages regenerate	Gel/Alg	Extrusion	Scaffold	Loaded cells	[79c]
	type I collagen	Extrusion	Hydrogel	Keratinocytes/melanocytes	[150a]
	Gel/Alg	Extrusion	Hydrogel	MSCs	[150b]
	PLCL	Extrusion	Scaffold	MFUS	[150c]
	Alg/Gel/PCL	Extrusion	Scaffold	SG cells	[151b]
	GelMA	In situ 3D printing	Hydrogel‐ scaffold	Zn/Si dual ions	[168]
Intelligent monitoring	SS/GelMA	Extrusion	Hydrogel‐ scaffold	SS	[13c]
	SA/PAM	Direct‐ink‐writing	Hydrogel	Ce^3+^/Ce^4+^	[60]
	DexG/Con A/PEGDMA/LAP	DLP	Hydrogel	Con A	[154]
	Alg	Co‐axial extrusion	Fiber	Mesoporous resin beads	[156]
ML‐assisted	Alg/DNA/DNA‐induced biosilica	Extrusion	Hydrogel	DNA/biosilica	[121]
	Wax	Inkjet	Sensor	AI‐enabled sensors	[158]
	HACC‐PAM	Alternative	Hydrogel	Colorimetric reagent	[159]
	PAM/LiCl/ N,N’‐methylenebisacrylamide/2‐hydroxy‐2‐methylpropiophenone/ethylene glycol	In situ 3D printing	Hydrogel‐ sensor	AI‐powered 3D printing system	[162]

#### 4.2.1 Hemostasis 3D Printing‐Based Hydrogel Dressings

Hemostasis occurs in the first stage of skin wound healing and plays a vital role in the process. Adhesive hydrogel can adhere to the wound for a long time and enrich hemostatic factors to promote hemostasis while physically blocking the wound from contact with the external environment.^[^ ^123^ ^]^ The adhesive properties of hydrogels can be achieved by physical interactions, such as electrostatic interaction or complexation, and chemical covalent binding, such as reactions between hydrogel surface active groups and tissue surface functional groups.

**Figure 7:**
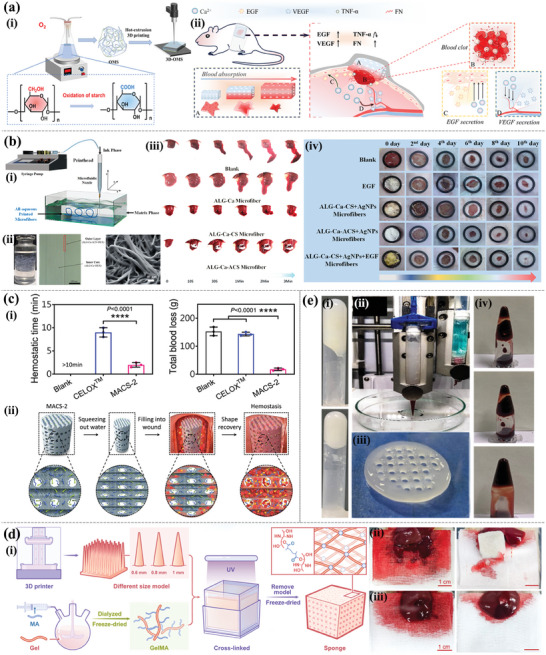
Hemostasis 3D printing‐based hydrogel dressings. a) Schematic illustration of 3D printing (i) and the mouse hemostatic evaluation (ii). Reproduced with permission.^[^ ^13a^ ^]^ Copyright 2023, Royal Society of Chemistry. b) Schematic illustrations of the all‐aqueous printing platform and printing process of microfibers (i). Optical microscopic and SEM images of the all‐aqueous printed microfibers (ii). Reproduced with permission.^[^ ^125^ ^]^ Copyright 2023, Elsevier. c) Hemostatic time and total blood loss in the blank, CELOXTM, and MACS‐2 groups (i). Schematic diagram of hemostatic procedure and mechanism of the MACS‐2 (ii). Reproduced with permission.^[^ ^126^ ^]^ Copyright 2021, Springer Nature. d) Preparation of microporous sponges with designed conical microchannels (i). The SD rat liver defect (ii) and penetration (iii) experiment. Reproduced with permission.^[^ ^127^ ^]^ Copyright 2023, American Chemical Society. e) Digital images of TCNF gel (i). Schematic illustration of the 3D printing process (ii). Digital images of 3D printed TCNF (iii) and blood coagulation (iv). Reproduced with permission.^[^ ^128^ ^]^ Copyright 2022, American Chemical Society.

Hydrogel hemostatic dressings can be printed in a variety of structural forms, among which the easiest approach is to modify the porous hydrogels themselves to enhance their hemostatic properties. As shown in **Figure** **7**a, Zheng et al.^[^ ^13a^ ^]^ constructed oxidized cornstarch hydrogel loaded with the coagulation factor Ca^2+^ using hot extrusion 3D printing technology and achieved rapid hemostatic wound effects as shown by modern analysis methods combined with in vivo and in vitro experiments. The strong adhesion of mussels to wet surfaces has inspired the development of a large number of hydrogel adhesives based on catechol groups.^[^ ^123^, ^124^ ^]^ Yang et al.^[^ ^124a^ ^]^ reported that a photo‐cross‐linked multi‐functional adhesive hemostatic hydrogel dressing based on functional CS, methacrylamide dopamine (DMA), and zinc ion showed good hemostatic ability in mouse liver hemorrhage and tail‐severed models. This dressing had combined antibacterial and antioxidant functions that promoted wound healing. The researchers optimized the structure of the hydrogel‐based materials and developed hemostatic dressings with fibers, stents, sponges, and other structures to further enhance their hemostatic effects. Xu et al.^[^ ^125^ ^]^ constructed bioactive microfibers by the 3D printing of SA, alkyl CS, and other mixed inks in a liquid environment (Figure 7b). The fiber size, contact area, contact point, and flow relationship between the microfiber and the blood can be adjusted to customize the microfiber size to the actual clinical conditions to achieve the best hemostatic performance. For the purpose of guiding the in situ tissue regeneration of incompressible hemorrhages in civil and battlefield environments, Du et al.^[^ ^126^ ^]^ modified the surface and optimized the structure of 3D printable microfibers to develop a hemostatic CS sponge with highly interconnected microchannels (microchannelled alkylated CS sponge) (Figure 7c). The sponge showed an excellent ability to promote coagulation and hemostasis in rat and pig liver perforation wound models, which is beneficial for treating fatal incompressible bleeding and promoting wound healing. In addition, considering the shape and structure design of the dressing, Cao et al.^[^ ^127^ ^]^ prepared GelMA porous sponges with a series of tapered microchannel structures using 3D printing and freeze‐drying techniques to further optimize the hemostatic performance of the sponge (Figure 7d). Experiments using SD rat liver defects and SD rat liver perforation wounds combined with theoretical model analysis showed that the taper (bottom diameter) and distribution of microchannels significantly affected the blood absorption and hemostatic ability of the sponge, which provided a new strategy for the development of high‐efficiency hemostatic dressing. Biranje et al.^[^ ^128^ ^]^ are interested in 3D‐printed scaffolds. They cross‐linked cellulose nanofibrils, CS, and casein in situ using green ion complexation to make customizable 3D scaffolds (Figure 7e). Human thrombin‐antithrombin showed that the 3D composite scaffolds could form stable blood clots with high thrombin content and accelerate blood clotting. The lower clotting index of the scaffolds represented higher coagulation efficiency induced by red blood cell encapsulation, indicating the great potential of 3D‐printed scaffolds in accelerating hemostasis.

Traditional methods (gauze or bandages, etc.) that rely on the body's own blood clotting system are associated with a risk of infection and have limited hemostatic effects.^[^ ^129^ ^]^ In contrast, adhesive 3D printing‐based hemostatic hydrogel dressings have better biocompatibility and biodegradability and can be customized to adapt to dynamic irregular wounds to meet complex clinical conditions and optimize hemostatic effects. Of note is that the swelling behavior of highly absorbent 3D printing‐based hydrogel dressings has a negative effect on tissue adhesion. Therefore, the wet surface adhesion and swelling ability of 3D printing‐based hemostatic hydrogel dressings need to be further coordinated and optimized.

#### 4.2.2 Anti‐Inflammatory 3D Printing‐Based Hydrogel Dressings

Inflammation occurs in the second stage of wound healing and is a defense response of the body. Proper inflammation can recruit immune cells to secrete cytokines and regulate the healing process. At the same time, the removal of injury factors and necrotic tissue is beneficial to maintaining the homeostasis of the wound microenvironment and promoting tissue repair and regeneration.^[^ ^130^ ^]^ However, when disturbed by external factors, the wound remains in the inflammation stage for a long time. This excessive inflammatory reaction will lead to oxidative stress, which can induce the sustained high expression of pro‐inflammatory factors accompanied by high levels of ROS, resulting in slow healing or the inability to heal.^[^ ^130a^ ^]^ Therefore, hydrogels with antioxidant and anti‐inflammatory functions are needed to capture or neutralize free radicals to reduce the expression of pro‐inflammatory factors, eliminate excessive ROS, and regulate wound healing.

**Figure 8:**
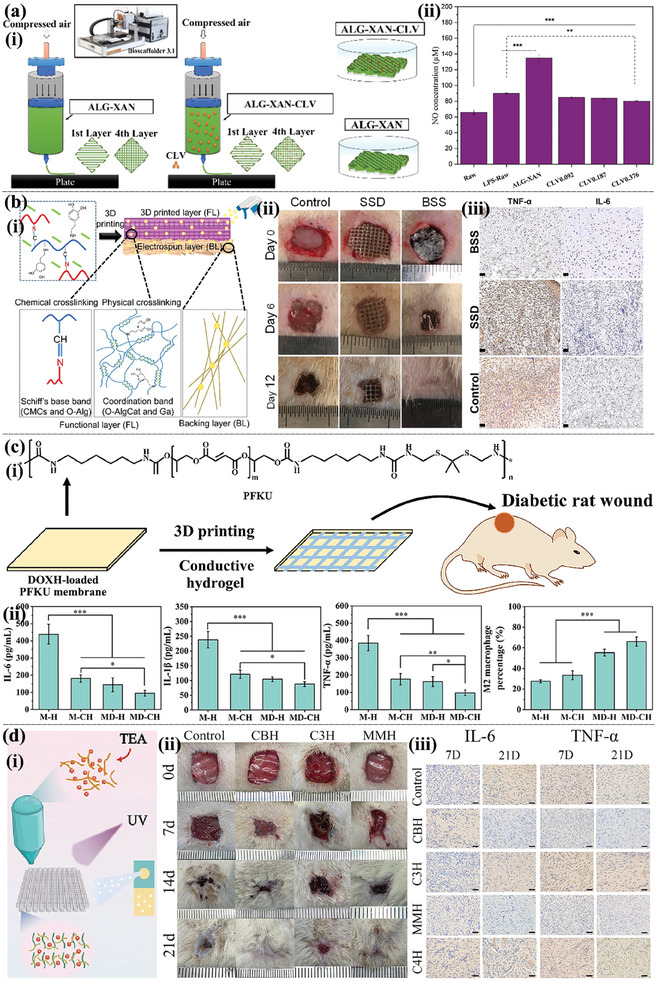
Anti‐inflammatory 3D printing‐based hydrogel dressings. a) Schematic illustration of the 3D‐printing process using ALG−XAN‐based biomaterial ink (i). The effects of hydrogels on NO production on LPS‐stimulated Raw 264.7 macrophage‐like cells (ii). Reproduced with permission.^[^ ^13b^ ^]^ Copyright 2023, American Chemical Society. b) Diagrammatic illustration of the multifunctional BSS's preparation approach and structure (i). In vivo infected burned wound healing studies of the BSS, commercial SSD and control (without treatment) (ii). Immunostaining of TNF‐α and IL‐6 on day 6 (scale bare = 100 µm) (iii). Reproduced with permission.^[^ ^132^ ^]^ Copyright 2023, Elsevier. c) Schematic diagram of composite dressing composed of conductive hydrogel strip and PFKU fiber membrane loaded with DOXH in the treatment of diabetic wound (i) and the concentrations of IL‐6, IL‐1 β, TNF‐ α, and polarization of M2 macrophages in wound tissues (ii). Reproduced with permission.^[^ ^134^ ^]^ Copyright 2022, Elsevier. d) In situ preparation of Ce‐based MOF hydrogels by 3D printing (i). The general view of the healing of animal wounds treated with different hydrogels for 0, 7, 14, and 21d (ii). The immunohistochemical staining of IL‐6 and TNF‐α of the healing tissue at 7 and 21 days. (Scale bar = 50 µm) (iii). Reproduced with permission.^[^ ^60^ ^]^ Copyright 2023, Elsevier.

Natural antioxidants or phytotherapeutic agents, such as tea polyphenols, aloe gel, curcumin, ferulic acid, and tree clove extracts,^[^ ^131^ ^]^ have been loaded into hydrogels to function as antioxidants in wound healing. Unalan et al.^[^ ^13b^ ^]^ developed 3D‐printed SA‐xanthan gum (ALG‐XAN) hydrogel loaded with clove essential oil (**Figure** **8**a). The experimental results showed that the addition of clove essential oil increased free radical scavenging ability, as well as the antioxidant and anti‐inflammatory activity of the hydrogel, indicating the potential of natural plant therapy in 3D printing‐based hydrogel dressings. Polymers containing catechol groups not only showed good adhesive and hemostatic ability but also effective anti‐inflammatory activity. Khalaji et al.^[^ ^132^ ^]^ used extrusion 3D printing technology to customize carboxymethyl CS (CMCs)/oxidized Alg grafted catechol (O‐AlgCat)‐based bioactive scaffolds grafted with catechol (Figure 8b). The dressing showed good anti‐inflammatory effects in an infected rat burn model, effectively reducing the expression of pro‐inflammatory factors and shortening the wound healing time. Zhu et al.^[^ ^133^ ^]^ prepared porous poly (L‐lactide/CS) (PLLA/CS) composite scaffolds with a micro/nanofiber layered structure using 3D printing and thermally induced phase separation technology, in which the poly layer (polydopamine, PDA) was functionalized by bioactive quercetin (Qu). The experimental results showed that the CS nanofiber network and Qu‐functionalized PDA promoted cell adhesion and proliferation and also demonstrated excellent cell affinity and anti‐inflammatory properties. In addition, the direct addition of anti‐inflammatory drugs or NPs to the hydrogel printing process can also be used as a convenient anti‐inflammatory agent, especially in diabetic wounds. Cao et al.^[^ ^134^ ^]^ printed GelMA and [2‐(acryloxy) ethyl] trimethylammonium chloride dressings on doxycycline hydrochloride (DOXH)‐supported polyurethane (PFKU) nanofiber films (Figure 8c). In vivo and in vitro experiments showed that the release of DOXH effectively downregulated the levels of ROS and inflammatory factors and promoted the polarization of macrophages to the M2 anti‐inflammatory phenotype, which is beneficial for accelerating the wound healing process. Nano‐enzymes have attracted a lot of attention and also have excellent anti‐inflammatory properties. Chen et al.^[^ ^60^ ^]^ developed a novel cerium‐based metal‐organic framework (MOF) nano‐enzyme hydrogel for the treatment of diabetic wounds (Figure 8d). In situ 3D printing technology not only improved the dispersibility of the nano‐enzyme but also enhanced the mechanical properties of the printed hydrogel. The prepared nano‐enzyme hydrogel displayed excellent scavenging ROS ability, thus reducing inflammatory reactions and promoting wound healing in diabetes.

Controlling and reducing excessive inflammation is essential for the tissue repair of diabetic and other chronic wounds. With the help of flexible 3D printing, anti‐inflammatory hydrogel‐based dressings can regulate ROS levels and macrophage polarization during wound healing, indicating the great potential of 3D printing‐based hydrogel dressings in guiding the immune system to promote chronic wound healing.

#### 4.2.3 Antibacterial 3D Printing‐Based Hydrogel Dressings

Bacterial infection is a common complication of the wound healing process and can cause persistent inflammatory reactions and slow the healing process.^[^ ^56^ ^]^ Although the current clinical use of antibiotics can effectively control bacterial infections, long‐term use and overuse can increase bacterial resistance, which is not conducive to effective wound treatment. Therefore, new and more effective antibacterial materials need to be developed.

**Figure 9:**
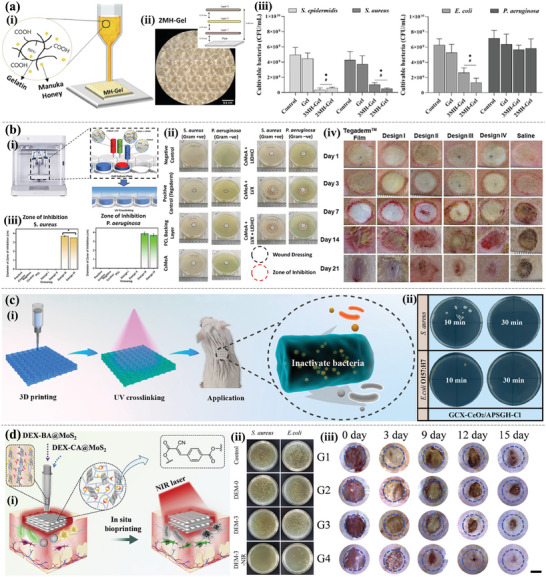
Antibacterial 3D printing‐based hydrogel dressings. a) Schematic illustration of the 3D‐printing process (i). Microscopic images obtained for 3‐layered 2MH‐Gel printed patches (ii) and antibacterial activity of 3D hydrogels against *S. epidermidis*, *S. aureus*, *E. coli* and *P. aeruginosa* after 24 h of incubation (iii). Reproduced with permission.^[^ ^135^ ^]^ Copyright 2022, Elsevier. b) Schematic of the fabrication of personalized CS methacrylate wound dressings (i). Evaluation of antimicrobial properties of different designs of CS methacrylate wound dressings, along with just the PCL backing layer (ii). Comparison of the zone of inhibition of each group for *S. aureus* and *P. aeruginosa* based on their respective diameters (iii). Digital images of the wound bed in all treatment groups (iv). Reproduced with permission.^[^ ^137^ ^]^ Copyright 2021, Wiley‐VCH. c) Schematic illustration of the fabrication and application of antibacterial 3D printed dressings (i). The pictures of agar plates of *S. aureus* and *E. coli* O157:H7 colonies contacted with dressings for 10 and 30 min (ii). Reproduced with permission.^[^ ^138^ ^]^ Copyright 2021, Elsevier. d) Schematic diagram of the fabrication of an MoS_2_ accelerated gelling hydrogel scaffold through microfluidic‐assisted in situ 3D printing technique (i). Photographs of bacterial colonies of *E. coli* and *S. aureus* after different treatments (ii). Images of wounds with different treatments on different days. Scale bar, 0.5 cm (iii). Reproduced with permission.^[^ ^139^ ^]^ Copyright 2023, Elsevier.

Natural antibacterial materials have attracted great attention because of their good biocompatibility. Brites et al.^[^ ^135^ ^]^ developed a natural antibacterial manuka honey (MH)/Gel 3D hydrogel patch based on the extrusion printing process (**Figure** **9**a), which not only promoted the proliferation of human dermal FBs and human epidermal keratinocytes but also demonstrated good antibacterial activity against gram‐positive bacteria [*Staphylococcus epidermidis* (*S. epidermidis*) and *Staphylococcus aureus* (*S. aureus*)] and gram‐negative bacteria (*Escherichia coli*, *E. coli*). Liang et al.^[^ ^136^ ^]^ successfully prepared 3D‐printed benzyl isothiocyanate (BITC) antibacterial hydrogel by introducing BITC into porous composite food hydrocolloids (e.g., XAN gum, locust bean gum, and carrageenan). The results showed that BITC‐XLCK‐Gel with 0.6% and 0.4% BITC had strong antibacterial activity against *S. aureus* and *E. coli*, respectively. In addition, like anti‐inflammatory hydrogel materials, adding antimicrobials or NPs in the printing process for slow release at the wound can also be an effective antibacterial method. Teoh et al.^[^ ^137^ ^]^ dispersed clinical drugs (such as lidocaine hydrochloride or levofloxacin) into CS/methacrylic anhydride gel using 3D printing and customized dressings of different shapes and sizes according to the wound (Figure 9b). A methacrylic acid CS‐drug sustained‐release gel dressing showed excellent antibacterial and anti‐infective effects in deep burn wound repair, thus promoting wound healing. Yang et al.^[^ ^138^ ^]^ prepared 3D‐printed dressings of CeO_2_/N‐halamine hybrid NPs/sodium CMC/XAN gum (Figure 9c). With the introduction of CeO_2_/N‐halamine biocompatible NPs, the dressing showed good inactivating effects on *S. aureus* and *E. coli*, which proved that the loading of inorganic metal oxide NPs could improve the antibacterial properties of the hydrogel.

Emerging photothermal antimicrobial therapy provides a promising alternative to traditional antibiotics because of its biosafety and ability to avoid drug resistance. Representative NIR response photothermal materials include PDA, graphene, and transition metal chalcogenides. Lu et al.^[^ ^56^ ^]^ used the extrusion 3D printing technique to prepare dopamine (DA) grafted Gel/SA/quaternary ammonium CS composite hydrogel. Antibacterial analysis showed that the antibacterial activity of 1.5% hydrogel against *S. aureus* and *E. coli* was 93.17% and 91.06%, respectively. Ding et al.^[^ ^139^ ^]^ designed a molybdenum disulfide (MoS_2_)‐accelerated gelling hydrogel scaffold to adapt to the shape of the wound using in situ 3D printing strategy (Figure 9d). The printed MoS_2_ hydrogel scaffold exhibited ROS scavenging and photothermal antibacterial effects, simultaneously playing roles in clearing bacterial infection, alleviating oxidative stress, providing oxygen, and significantly promoting the healing of diabetes‐associated chronic infections.

3D printing‐based hydrogel materials with inherent antibacterial properties can maintain continuous antibacterial activity in the wound environment; however, their antibacterial activity is relatively weak. Antimicrobial agents (e.g., drugs, antimicrobial peptides, or NPs) released from 3D printing‐based hydrogel dressings have sustained antibacterial ability and show good antibacterial wound effects.^[^ ^137^, ^138^ ^]^ Additionally, emerging photothermal antibacterial therapy can achieve controllable antibacterial effects and is less invasive with fewer side effects. However, 3D printing‐based photothermal antibacterial hydrogel dressings have the problem of excessively high temperatures during the illumination process, so the selection of illumination parameters, the photothermal materials loaded, and their concentrations need to be further optimized.

#### 4.2.4 Self‐Healing 3D Printing‐Based Hydrogel Dressings

The physical stress, mechanical load, and external tension produced in the process of skin tissue activity deform or break the hydrogel, resulting in the loss of its structure and function, which adversely affects wound healing.^[^ ^140^ ^]^ Similar to the ability of human skin to repair itself after failure, self‐healing hydrogel can repair mechanical damage, maintain an intact structure, and restore the original characteristics, which is more suitable for applications in complex and changeable environments.

**Figure 10:**
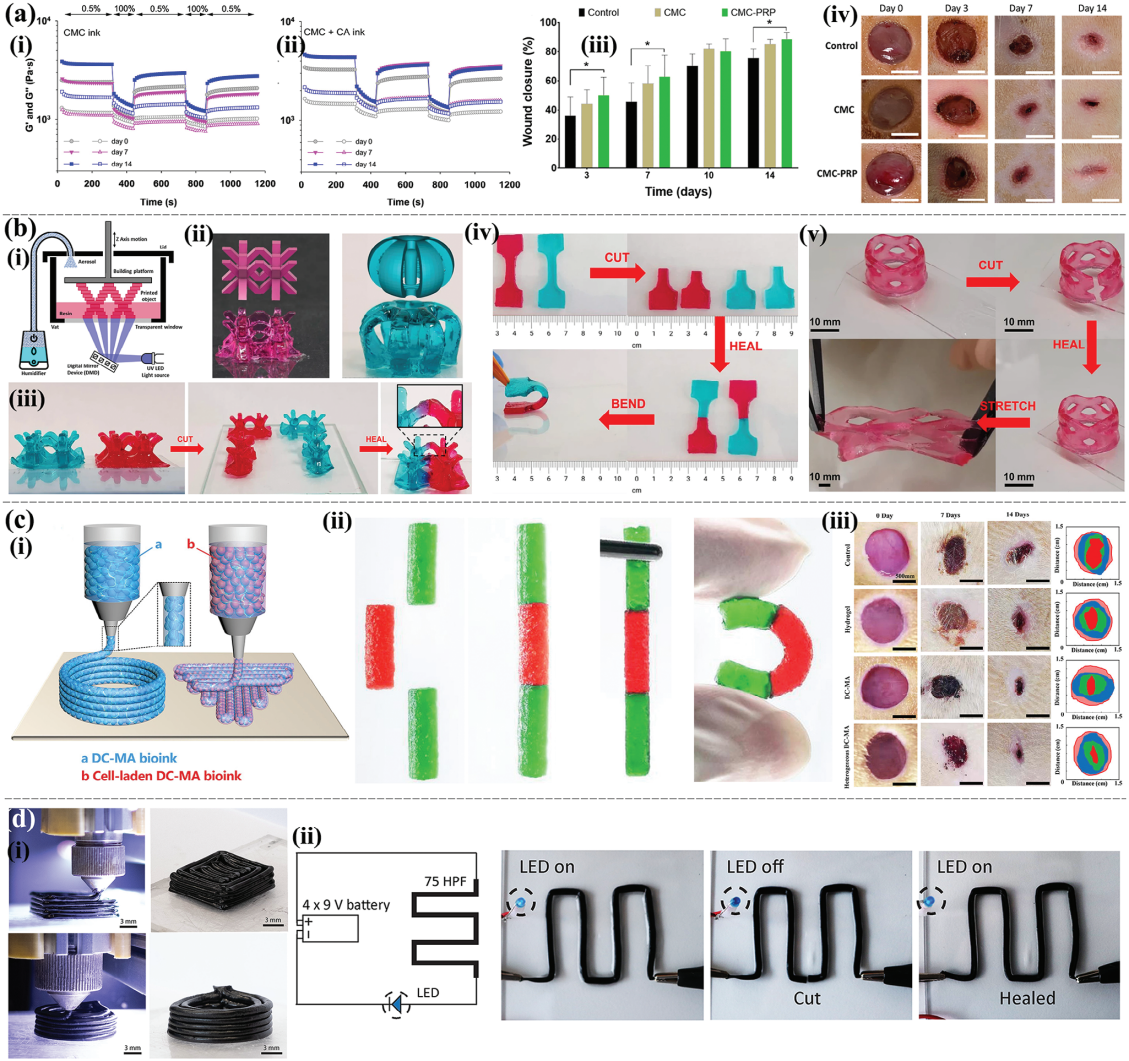
Self‐healing 3D printing‐based hydrogel dressings. a) Rheological properties of CMC dispersions with and without CA as freshly prepared and after storage under cyclic changes in shear strain (0.5% –100%) (i). Contraction of wounds (ii). Representative images of wound healing. Scale bar: 5 mm (iii). Reproduced with permission.^[^ ^87c^ ^]^ Copyright 2022, Elsevier. b) Schematic overview of the DLP printing (i). 3D fabricated samples (ii) and the restoration of cut‐rejoined objects printed with a ratio of AAc/PVA of 0.8 (iii‐v). Reproduced with permission.^[^ ^142^ ^]^ Copyright 2021, Springer Nature. c) Printability of DC‐MA bioink (i). Self‐healing of DC‐MA rods (ii). Gross observation of the wounds treated with normal saline, hydrogel, DC‐MA, and heterogeneous DC‐MA (iii). Reproduced with permission.^[^ ^143^ ^]^ Copyright 2022, American Chemical Society. d) Pictures of extrusion 3D printing (i) and the schematic of the electrical circuit generated through cut or healed Gum (ii). Reproduced with permission.^[^ ^144^ ^]^ Copyright 2021, American Chemical Society.

Self‐healing hydrogels mainly carry out self‐healing functions through physical self‐repair (such as hydrogen bonding, electrostatic attraction, and hydrophobic interaction) and chemical self‐repair (dynamic covalent bond).^[^ ^140a^ ^]^ Gomez et al.^[^ ^87c^ ^]^ screened the printability and fidelity of aseptic, concentrated natural CMC/citric acid (CA) dispersions produced by extrusion 3D printing. Suitable concentrations of CMC ink showed excellent self‐healing rheological properties and stability (**Figure** **10**a). Distinct from gel containing a small amount of CMC, scaffold printed with sufficient concentrations of CMC ink fully absorbed exudate and increased cell fluidity to accelerate diabetic wound healing. Isik et al.^[^ ^141^ ^]^ are interested in all‐natural self‐healing hydrogels and have conducted related research. SA, tyramine‐modified HA, and decellularized bovine aorta ECM were rapidly cross‐linked in situ using extrusion 3D printing. The composite hydrogels had high elasticity, self‐recovery properties, and excellent biological activity, which have potential applications in regenerative medicine. In addition to conventional extrusion printing technology, printing self‐healing hydrogel using photopolymerization technology has also been developed. Caprioli et al.^[^ ^142^ ^]^ prepared semi‐interpenetrating polymer hydrogels with complex 3D structures from commercially available compound materials, such as PVA and acrylic acid (AAc), and light‐cured the substances using commercial DLP printers (Figure 10b), which achieved self‐healing without any stimulation or external triggers and recovered 72% of its initial strength after 12 h, reducing processing costs while improving printing resolution. DA polymers are also widely used in the field of self‐healing hydrogels because of their ability to chelate polyphenols. Feng et al.^[^ ^143^ ^]^ proposed a new dynamic cross‐linking strategy to meet the challenge of the contradiction between the printability/shape fidelity and the cell viability of hydrogel materials, as shown in Figure 10c. DA‐modified HA was used as a dynamic cross‐linking agent to assemble biological links with carrier cell hydrogels [hyaluronic acid‐modified methacrylate (HAMA)‐phenylboric acid (PBA)/GelMA], which not only ensured high printability and cell viability of the materials, but also improved the microporosity, tissue adhesion, and self‐healing of the 3D‐printed hydrogels, which was beneficial to accelerating the wound healing process. Pierchala et al.^[^ ^144^ ^]^ designed a composite polymer hydrogel based on halloysite nanotubes (HNT), PDA, iron ions (Fe^3+^), and PVA (Figure 10d). The arrangement of PDA‐modified high stone nanotubes improved the strain capacity, mechanical stiffness, and electrical conductivity of the composites. More importantly, it achieved rapid self‐healing in a few seconds. The self‐healing hydrogel can be customized into electronic skin material suitable for tissue engineering using 3D printing technology, which has a broad application prospect in the field of electrical‐stimulated wound healing and wound monitoring.

One of the essential characteristics of biomaterials for long‐term application is mechanical durability. The integrity of the hydrogel network structure is likely to be damaged by external forces, reducing its therapeutic effects. 3D‐printed hydrogel material with self‐repairing ability can achieve reversible network dynamic linkage to restore the original structure and function.^[^ ^140b^ ^]^ However, few reports have specified the stability of the long‐term self‐healing properties of 3D‐printed self‐healing hydrogels under physiological conditions. Especially because of the complex wound microenvironment, further research on the long‐term reliability of chemical bonds affected by pH, enzymes, and various microorganisms is needed. In addition, the mechanical strength of 3D printing‐based self‐healing hydrogel dressings is generally insufficient, so additional studies are needed to improve the mechanical properties while ensuring biocompatibility and durability.

#### 4.2.5 Stimulus‐Responsive Drug Release 3D Printing‐Based Hydrogel Dressings

Porous‐structure hydrogel, which can be transported to a specific location, is suitable for loading various types of drug molecules and functional factors. The dose can be controlled in the presence of precursors that maintain the stable activity of biological factors to achieve accurate and sustained release.^[^ ^28^ ^]^ Multiple stimulus‐responsive hydrogels can respond to subtle changes in physical, chemical, and biological conditions, such as light, electricity, magnetism, temperature, and pH, and are commonly used as carriers to control drug delivery.^[^ ^28^, ^145^ ^]^

**Figure 11:**
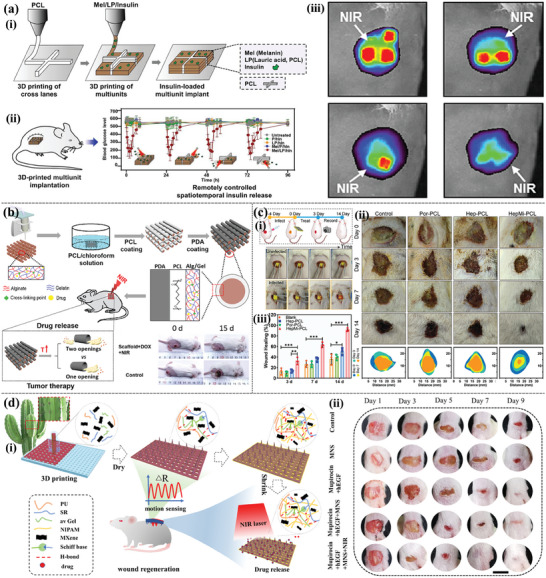
Stimulus‐responsive drug release 3D printing‐based hydrogel dressings. a) Schematic illustration of the Mel/LP/hIn implant printing process (i). Mice implanted with the various 3D‐printed implants were irradiated with NIR at a different locus once a day for four days and the blood glucose levels were monitored for 96 h after the first NIR irradiation (*n *= 5) (ii). The in vivo light‐responsive fhIn release from Mel/LA/PCL/fhIn (iii). Reproduced with permission.^[^ ^33^ ^]^ Copyright 2022, Elsevier. b) The scheme of preparation of PDA/Gel/PCL core/shell scaffolds with NIR triggered on demand drug release for tumor therapy and wound healing. Reproduced with permission.^[^ ^147^ ^]^ Copyright 2021, Elsevier. c) Treatment schedule for the infection‐induced chronic wounds of rats and visualization of infection within tissues as an indicator by HepMi‐PCL (i). Gross inspection (ii) and the ratio of wound healing (iii) at 3, 7, and 14 days. Reproduced with permission.^[^ ^58^ ^]^ Copyright 2024, Wiley‐VCH. d) Schematic illustration of 3D‐printed bionic NIR light‐responsive MXene and spidroin‐based microneedle scaffolds for skin wound healing (i). HE staining of wounds in mice; scale bar: 100 µm (ii). Reproduced with permission.^[^ ^148^ ^]^ Copyright 2022, American Chemical Society.

Baykara et al.^[^ ^146^ ^]^ designed and manufactured a wound dressing scaffold loaded with the antibiotic drug amoxicillin (AMX), which was made of a 3D‐printed CS biological link doped with bismuth ferrite (BFO) NPs and had good tensile strength and cytocompatibility. In particular, the ferroelectricity of BFO NPs enabled the dressings to release AMX under mild electrical stimulation in vitro, indicating the potential of dressings to deliver therapeutic drugs in a controlled manner. In addition, photostimulation, especially photothermal, is an effective way to promote drug release. Kim et al.^[^ ^33^ ^]^ constructed PCL/lauric acid/melanin (PCL/LA/Mel) scaffolds loaded with insulin using 3D printing (**Figure** **11**a). Under NIR irradiation, the heat produced by Mel dissolves the scaffold's matrix and releases insulin from the inside, indicating that this strategy can achieve multi‐dose therapy under NIR stimulation. As a representative photothermal material, PDA is also widely used for drug release induced by light. As shown in Figure 11b, Liu et al.^[^ ^147^ ^]^ generated PDA‐coated Alg‐Gel/PCL core/shell scaffolds to optimize the photothermal effect on the scaffolds. Triggered by NIR light, the heat generated by PDA induced the sol‐gel transition of the core gel, thus achieving the release of a drug (adriamycin) on demand, providing a strategy with great application potential in repairing tissue defects and promoting tissue regeneration.

The emerging porous microneedle patch has also been shown to respond to wound‐related biomarkers in real‐time and stimulate drug release to promote wound healing, achieving both diagnostic and therapeutic effects. Liu et al.^[^ ^58^ ^]^ developed a biomimetic PCL microneedle patch filled with heparin hydrogel based on high‐precision 3D printing technology (Figure 11c), which controlled infections and exhibited drug‐controlled release. A microneedle patch can automatically change the speed of drug delivery according to the amount of pus and achieve independent drug administration on demand, thus increasing the healing speed of infected wounds in mice by more than 200%.^[^ ^58^ ^]^ Shao et al.^[^ ^148^ ^]^ used hydrogel precursors composed of biocompatible water‐soluble PFKU, aloe vera gel, and spider solution mixed with MXene to obtain integrated microneedle scaffolds using extrusion 3D printing technology (Figure 11d). The temperature‐responsive n‐isopropylacrylamide drug‐loaded gel further permeated the interstitial space of the stent. Taking advantage of the excellent photothermal properties of MXene, the composite gel microneedle was shown to regulate drug release in response to infrared light, thus promoting wound healing. Therefore, the integration of diagnosis and treatment in 3D‐printed microneedles is expected to provide a new method for managing chronic, infected wounds.

Biomaterials that can sense external stimuli (e.g., light, electricity, temperature, glucose, and ROS) can be induced by external energy to release therapeutic drugs.^[^ ^28^ ^]^ Therefore, 3D‐printed hydrogel dressings for stimulus‐responsive drug release can release the corresponding loaded drugs according to various environmental changes, thus controlling the process of wound healing. An organism is a complex system that can produce different forms of stimulation at the same time. Therefore, multi‐stimulation‐sensitive 3D printing‐based hydrogel dressings that can respond to different wound environments simultaneously are expected to become a development direction in the future.

#### 4.2.6 Skin Appendages Regenerated 3D Printing‐Based Hydrogel Dressings

The overactivation of immune cells and the inflammatory reaction led to different degrees of fibrosis in the process of deep wound repair, resulting in an incomplete structure and non‐functional healing. For example, cicatricial non‐functional healing excluding hair follicles (HFs), sweat glands, sebaceous glands, and other appendages not only affects appearance and function but can cause psychological disorders in patients and affect their quality of life.^[^ ^149^ ^]^ 3D printing‐based hydrogel dressings composed of biomaterials, active factors, and cells have been found to have the potential to promote the regeneration of various appendages.^[^ ^79^, ^150^ ^]^

**Figure 12:**
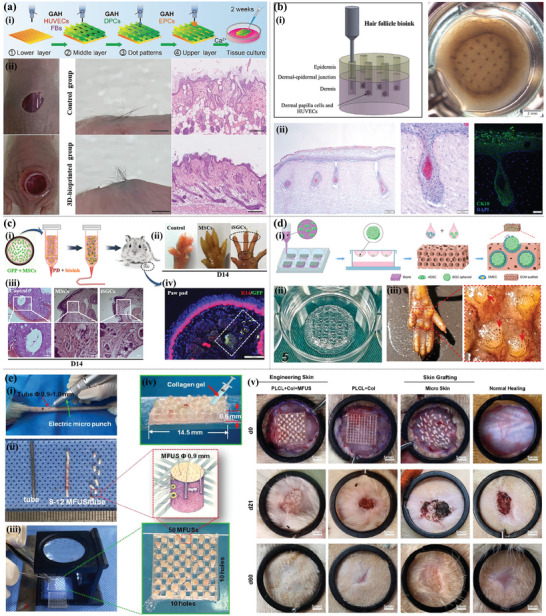
Skin appendages regenerated 3D printing‐based hydrogel dressings. a) Schematic workflow of the 3D bioprinting procedure(i) and 3D‐bioprinted multilayer composite scaffolds for HFs reconstruction in vivo (ii). Reproduced with permission.^[^ ^79c^ ^]^ Copyright 2022, Elsevier. b) Schematic of the strategy for printing hair follicle structures within the reconstructed skin models and live image of representative skin model in culture at day 2 (i). Histological analysis and immunofluorescence of 3D bioprinted skin models incorporating HF (ii). Reproduced with permission.^[^ ^150a^ ^]^ Copyright 2023, American Association for the Advancement of Science. c) Schematic illustration of approaches for engineering induced sweat glands (iSGCs) and transplantation (i). Sweat test of mice treated with different cells (ii). Histology of plantar region without treatment and transplantation (scale bars, 200 µm) (iii). Involvement of GFP‐labeled iSGCs in directed regeneration of SG tissue in thermal‐injured mouse model (K14, red; GFP, green; DAPI, blue; scale bar, 200 µm) (iv). Reproduced with permission.^[^ ^150b^ ^]^ Copyright 2020, American Association for the Advancement of Science. d) Schematic showing establishment of a biomimetic SVIM in vitro (i). Overview of the 3D‐bioprinted ADSC‐loaded constructs (ii). Sweat test of mice treated with iSGCs spheroids (iii). Reproduced with permission.^[^ ^151b^ ^]^ Copyright 2022, Elsevier. e) Photo image showed the MFUSs were harvested from rat tail skin using an electric micro puncher (i), the 8–12 MFUSs harvested and stored in a tube (ii) and the MFUS were loaded into the 3D‐printed PLCL scaffold using a metal stick under a microscope (iii). Photo image of tissue engineering skin formed by MFUS‐loaded 3D‐printed PLCL scaffold with collagen gel (iv). Wound healing of full‐thickness rat dorsal skin defects with different treatments at different time points (v). Reproduced with permission.^[^ ^150c^ ^]^ Copyright 2022, Sage Publications.

Kang et al.^[^ ^79c^ ^]^ encapsulated FBs, human umbilical vein endothelial cells (HUVECs), dermal papilla cells (DPCs), and EPCs in 3D‐printed Gel/Alg gel to construct a multilayer composite scaffold simulating the HF microenvironment in vivo, which promoted the formation of self‐aggregating DPC spheres and restored the expression of genes (ALP, β‐catenin, and α‐SMA) related to hair induction (**Figure** **12**a). As a result, immature HFs were successfully self‐assembled in vitro. Catarino et al.^[^ ^150a^ ^]^ also reported the inclusion of HF appendages in engineered skin tissue using 3D printing (Figure 12b). Printing induced DPCs and HUVECs into spheres, and then accurately printed in the gel dermis containing FBs. The mature tissue developed an HF structure, with morphology and composition similar to those of natural skin tissue, which had a significant impact on regenerative medicine. Apart from HFs, sweating is an important way for mammals to regulate body temperature, so it is also significant for the functional reconstruction of sweat glands in wound tissue. Fu et al. conducted a series of representative studies on the reconstruction of sweat glands.^[^ ^150^, ^151^ ^]^ Yao et al.^[^ ^150b^ ^]^ printed a sweat gland‐like matrix to guide the transformation of mesenchymal stem cells (MSCs) into functional sweat glands and promote the recovery of sweat glands in the soles of burned mice (Figure 12c). Further analysis of differential ECM protein expression showed that CTHRC1 and Hmox1 could promote the gene expression profile of sweat glands, which provided a theoretical basis for exploring the effect of a 3D‐printed matrix on the entry of MSCs into the gland lineage and the recovery of functional sweat glands. Yuan et al.^[^ ^151b^ ^]^ in the same group directed adipose‐derived mesenchymal stem cells to differentiate into sweat gland cell spheres in a 3D printing ECM microenvironment and then cultured them in vascular niches made of dermal microvascular endothelial cells, which promoted the occurrence of physiologically related vascularized glands, indicating the ability of vascularization to promote natural sweat gland regeneration and providing a new idea for sweat gland reconstruction in the process of wound tissue repair (Figure 12d). In addition, sebaceous glands, which widely exist in healthy skin, play an important role in regulating normal skin function, including moisturizing skin to prevent water loss, the skin's immune response, and barrier protection.^[^ ^152^ ^]^ Therefore, the reconstruction of sebaceous glands is particularly important for the functional regeneration of wound tissue. Chang et al.^[^ ^150c^ ^]^ demonstrated a new type of tissue engineering functional skin. A 3D‐printed polylactic acid‐co‐caprolactone scaffold and collagen gel loaded with the minimum functional unit of rat autologous tail skin (**MFUS**) were designed to reduce the risk of contamination in artificial skin compared to traditional allografts (Figure 12e). The results showed that the 3D‐printed sample induced the migration of skin FBs and promoted the regeneration of full‐thickness skin with HFs and sebaceous glands.

The ultimate goal of wound tissue repair is to restore the complete structure and function of the skin. The skin substitute reconstructed by traditional tissue engineering is limited by structural instability and the complexity of physiological properties, so it cannot deeply restore the natural skin of the organism.^[^ ^150c^ ^]^ The emerging advanced 3D printing technology uses a single automated platform to realize the spatiotemporal integration of multiple types of skin cells and biomaterials to build fully functional skin with necessary skin appendages (HFs, sweat glands, and sebaceous glands), thus overcoming the current limitations of tissue engineering and accelerating the progression of the skin structure.

#### 4.2.7 Intelligent Monitoring 3D Printing‐Based Hydrogel Dressings

The parameters near the wound will change dynamically with different healing stages. Therefore, the development of intelligent dressings that can sense the condition of the wound in real‐time, which can be used to adjust the treatment plan according to the feedback information, will provide more favorable conditions for wound repair with accurate treatment and eventually achieve the best wound healing effect.^[^ ^153^ ^]^

**Figure 13:**
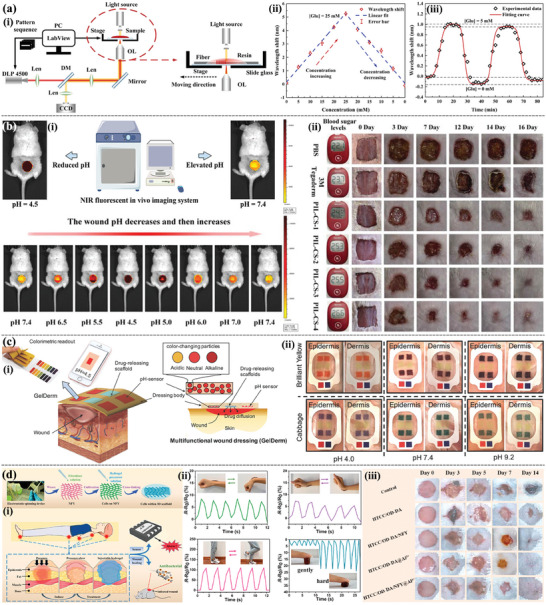
Intelligent monitoring 3D printing‐based hydrogel dressings. a) Setup for fabricating the LPG glucose sensor (i). Wavelength shift with increase and decreasing in glucose concentration and wavelength shift with time in the range of glucose concentration of 0–5 Mm (ii). Reproduced with permission.^[^ ^154^ ^]^ Copyright 2023, Elsevier. b) Real‐time imaging schematic of NIR fluorescent hydrogel PIL‐CS on in vivo diabetic mice at different pH levels (i) and representative pictures of wounds on days 0, 3, 7, 12, 14, and 16 (ii). Reproduced with permission.^[^ ^155^ ^]^ Copyright 2023, Wiley‐VCH. c) Schematic representation of GelDerm treatment of epidermal wounds with pH‐sensitive and drug‐eluting components (i). Images of GelDerm/Mepitel dressings placed on the dermis and epidermis layers of pig skin sprayed with different pH values (ii). Reproduced with permission.^[^ ^156^ ^]^ Copyright 2017, Wiley‐VCH. d) Illustration of the fabrication of a pressure monitoring 3D hybrid scaffold with aligned NFY embedded in injectable hydrogels (i). Relative resistance changes for hydrogels strain sensors under various bending conditions and the amplitude of corresponding signals for different levels of pressure (ii). Photos of infected wound closure with different treatments (iii). Reproduced with permission.^[^ ^122^ ^]^ Copyright 2022, Elsevier.

Traditional wound dressings, such as gauze, sponge, and cotton wool, are nearly opaque and need to be exposed to the wound frequently, which has an adverse effect on wound healing. To address this problem, Chen et al.^[13c]^ developed a natural silk glue‐based transparent interpenetrating network hydrogel scaffold made of silk sericin (SS) and GelMA using 3D printing. The scaffold showed good cytocompatibility and was beneficial to cell adhesion and proliferation. More importantly, transparent hydrogel has a good visual wound nursing effect, which provides the basic conditions for the realization of real‐time wound monitoring and nursing. As the wounds of patients with diabetes are likely to develop into chronic wounds, Wei et al.^[^ ^154^ ^]^ designed UV‐curable hydrogels based on glycidyl methacrylate‐modified dextran (DExG)/concanavalin A (Con A)/Poly(ethylene glycol) diamethacrylate (PEGDMA), as shown in **Figure** **13**a. A polymeric hydrogel grating with a micro‐scale structure was fabricated on the fiber surface using micro‐3D printing technology. This could selectively combine with glucose molecules and monitor blood glucose concentrations in real‐time through shifts in the transmission wavelength. Studies have shown that the pH value of the wound microenvironment changes significantly during wound healing, so monitoring the pH is reliable and effective. Zong et al.^[^ ^155^ ^]^ developed a multi‐functional composite hydrogel using polymerized ionic liquid (PIL), a NIR fluorescence probe, and CS (PIL‐CS), which had good conductivity, hemostasis, antibacterial activity, biocompatibility, and the ability to promote the production of VEGF. In particular, the composite hydrogel could respond to dynamic pH changes in a variety of wound microenvironments and, accordingly, continuously release drugs, such as antioxidants, to promote the wound healing process (Figure 13b), making it suitable for real‐time wound monitoring, skin repair, and regeneration in diabetes patients. Mirani et al.^[^ ^156^ ^]^ proposed an advanced multi‐functional Alg dressing loaded with antibiotics (gentamicin sulfate) to improve the intelligent effect of dressings. The dressing had the ability to measure pH values colorimetrically and detect bacterial infections while releasing drugs on the wound (Figure 13c). Further integrating the dressing onto a commercial patch and connecting it to a smartphone through a wireless interface can facilitate the transmission of digital image information of the wound between patients and medical staff. As a kind of intelligent dressing, it has broad prospects for treating acute and chronic wounds. Additionally, Qiu et al.^[^ ^122^ ^]^ conducted the real‐time monitoring of wound pressure sensing from the perspective of preventing PU (Figure 13d). NFY woven with PAN and rGO were encapsulated in injectable 3D hydrogel scaffolds, which showed excellent cytocompatibility, self‐healing ability, and broad‐spectrum antibacterial activity. Al^3+^ contained in the stent enhanced the electrical conductivity of the hydrogel and could be used as a wound pressure monitoring sensor to control the cellular direction and promote wound healing.

Intelligent monitoring 3D printing‐based hydrogel dressings can monitor the state of wounds in real‐time and make visual presentations in various media through integrated biochemical sensor detection units and data transmission systems, which is conducive for use in remote medical services and in promoting efficient wound management.^[^ ^154^, ^156^ ^]^ Since the microenvironment of complex wounds (such as diabetic wounds) is affected by multiple factors, the sensitivity and accuracy of multi‐index intelligent monitoring systems need to be improved. However, it is undeniable that intelligent monitoring 3D printing‐based hydrogel dressings remain an important development direction in wound dressings.

#### 4.2.8 Machine Learning‐Assisted 3D Printing‐Based Hydrogel Dressings

Machine learning (ML) uses the model parameters learned by a computer from training data to analyze and accurately predict the experimental data. As one of the core research projects of artificial intelligence (AI), ML has been successfully applied in the fields of Internet, automation, criminal investigation, and others. Especially for biomedicine, ML provides a more accurate technical approach for disease diagnosis, gene sequence analysis, and protein structure predictions.^[^ ^157^ ^]^

**Figure 14:**
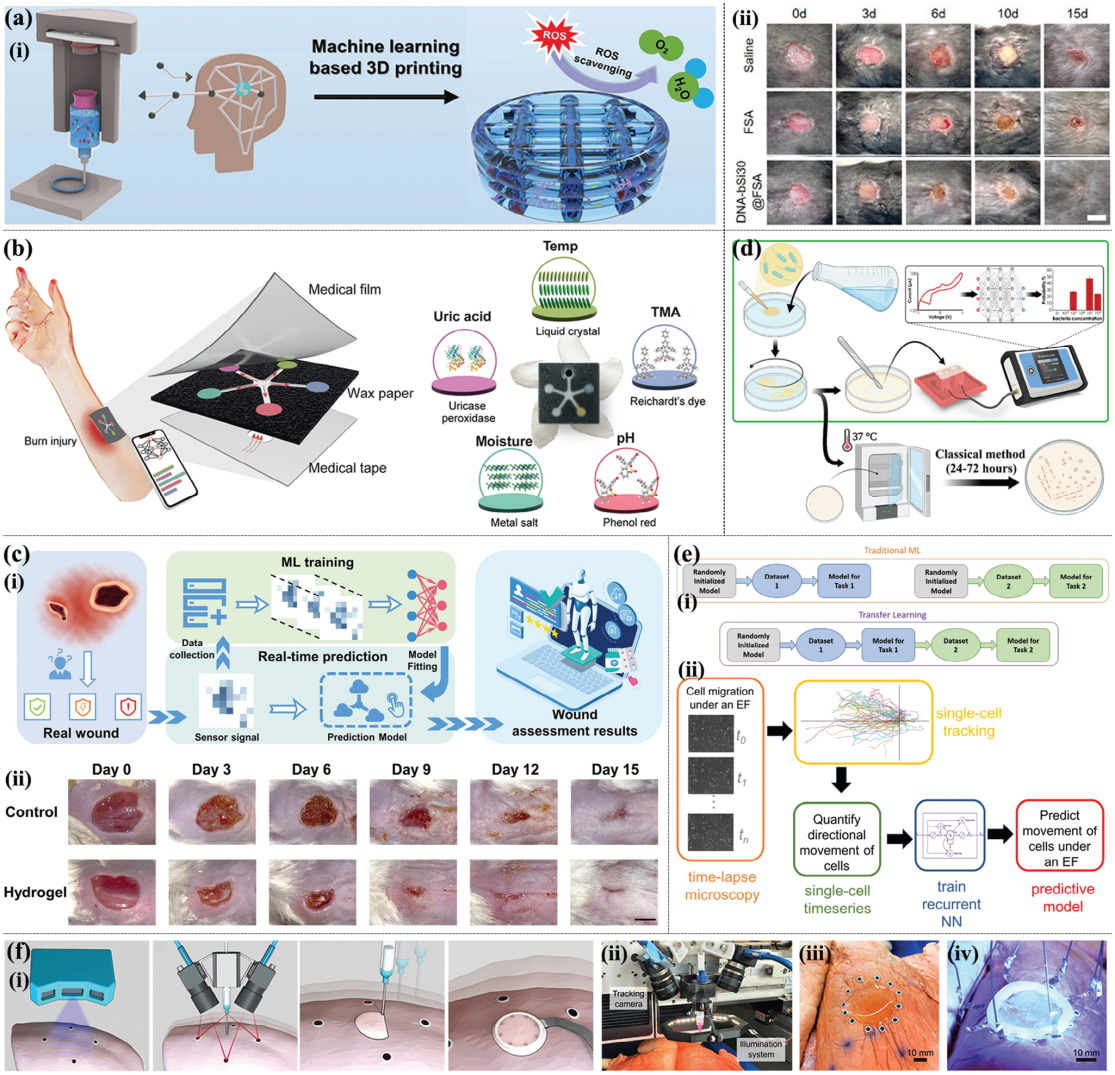
ML‐assisted 3D printing‐based hydrogel dressings. a) Schematic diagram of the fabrication of ML‐based 3D‐printed hydrogels (i). Captured images of the wound area after the determined breeding time (ii). Reproduced with permission.^[^ ^121^ ^]^ Copyright 2023, Wiley‐VCH. b) Illustration of the PETAL sensor adhered onto a burn wound for colorimetric analysis of wound healing status with the detailed LBL structure of the PETAL sensor and the sensing materials/principles for each colorimetric sensor. Reproduced with permission.^[^ ^158^ ^]^ Copyright 2023, American Association for the Advancement of Science. c) The process flows of personalized wound management from sensor signal collection to ML training and real‐time prediction (i). Representative photographs of skin wound treated with multifunctional hydrogel dressing. Scale bar: 5 mm (ii). Reproduced with permission.^[^ ^159^ ^]^ Copyright 2022, Elsevier. d) Brief scheme of the ML‐assisted electrochemical platform for determining the concentration of bacteria in comparison with the classical method. Reproduced with permission.^[^ ^160^ ^]^ Copyright 2022, American Chemical Society. e) Diagram comparing the traditional ML training approach with the transfer learning approach (i). The image processing and cell directedness prediction pipeline (ii). Reproduced with permission.^[^ ^161^ ^]^ Copyright 2022, Springer Nature. f) Process of in situ 3D printing of EIT sensor on a breathing lung (i). Photograph of the custom‐built 3D printing gantry system (ii). The 3D printed circular layer of hydrogel (iii). UV light curing of the hydrogel layer with the silicone ring and embedded electrodes (iv). Reproduced with permission.^[^ ^162^ ^]^ Copyright 2020, American Association for the Advancement of Science.

ML shows the great potential of AI in wound healing. Kim et al.^[^ ^121^ ^]^ found that hydrogel dressings showed good accuracy, shape fidelity, and mechanical adjustment in the 3D printing process optimized by ML methods and could provide customized dressings for the effective treatment of wounds with different shapes and depths (**Figure** **14**a). Zheng et al.^[^ ^158^ ^]^ reported a paper‐like battery‐free in situ AI‐enabled multiplexer (PETAL) sensor that could respond to temperature, pH, trimethylamine, uric acid, and moisture (Figure 14b). A deep learning algorithm, one of the most popular branches of ML, was used to monitor and evaluate wound healing in situ, with an accuracy as high as 97%. Wang et al.^[^ ^159^ ^]^ also designed a personalized wound management model based on a convolution neural network learning algorithm, which is a deep learning technique (Figure 14c). Then, the colorimetric signal of a hydrogel dressing (polyacrylamide and chitosan quaternary ammonium salt, HACC‐PAM) was measured to evaluate wound healing and infection, with an accuracy of 94.47%. This research provided an advanced solution for intelligent wound monitoring and took an inestimable step toward future intelligent wound management. In addition, ML has assisted in other interesting research endeavors. Lavrentev et al.^[^ ^160^ ^]^ developed a bio‐electrochemical platform based on a soft hydrogel/eutectic gallium‐indium alloy interface (Figure 14d), in which the multilayer perceptron model could detect bacterial concentrations in culture with a high accuracy (94%), providing new ideas for improving the antibacterial function of dressings in the wound healing process. Sargent et al.^[^ ^161^ ^]^ proposed an ML model that could predict the direction of cell migration under an electric field based on previous time series of directionality and electric field values (Figure 14e). Then, a proportional‐integral‐derivative controller was employed to simulate the feedback control of cell migration, which, in turn, verified the predicted results of a long short‐term memory model. This data‐driven strategy provides a powerful scientific basis for the precise regulation of cells using an electric field to promote wound healing. Additionally, Zhu et al.^[^ ^162^ ^]^ developed a new AI‐powered on‐site 3D printing system with an electrical impedance tomography (EIT) sensor to enrich 3D printing technology. Based on “offline” ML and an “online” computer vision tracking algorithm, the system changes the printing trajectory in real time following the motion and deformation mapping of the target surface to realize the adaptive printing of wearable biomaterials on and inside the human body (Figure 14f). In situ autonomous 3D printing technology can achieve accurate spatial control for a long time in the printing process of biomaterials, such as engineered cell scaffolds, surgical glue, and skin transplantation for tissue regeneration, which is helpful for stimulating surgical robot technology in patient monitoring and wound treatment.

Different from the traditional statistical model, ML has the ability to actively learn complex relationships between transmitted data and accurately predict and evaluate the results. Therefore, the introduction of ML‐assisted 3D printing‐based hydrogel dressings is expected to reduce the human error rate in wound management and improve medical accuracy. Only a few studies have studied ML, 3D printing hydrogel, and wound healing together.^[^ ^121^, ^160^, ^161^ ^]^ However, the results indicated that ML‐assisted 3D printing‐based hydrogel dressings have great potential in the field of precision medicine.

## 5 Clinical Application Prospects of 3D Printing‐Based Hydrogel Dressings

**Figure 15:**
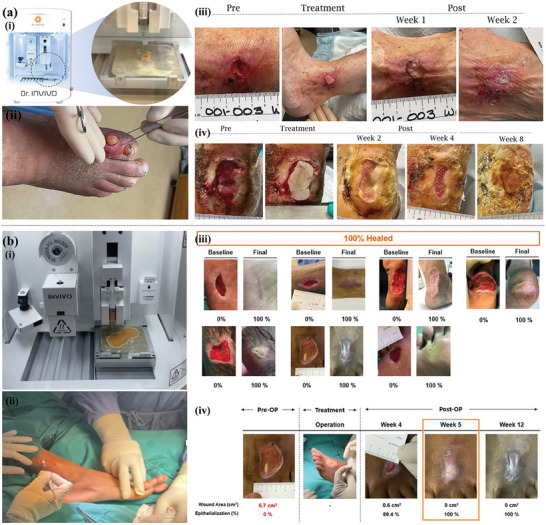
Clinical case of treating diabetic foot with 3D printing‐based gel dressings. (a) 3D bioprinting system for AMHAT (i); Single treatment of AMHAT (ii); Case example showing a 66‐year‐old woman with chronic DFU on lateral ankle (iii); Case example shows a 58‐year‐old man with chronic heel DFU(iv). Reproduced with permission.^[^ ^169a^ ^]^ Copyright 2022, Wolters Kluwer Health, Inc. on behalf of The American Society of Plastic Surgeons. (b) Photograph of the procedure: Dispensing of fat tissue according to the wound size (i); Application of the dispensed 3D‐AMHAT onto the wound (ii); Corresponding wound pictures taken from the study (iii); Wound healing process in a patient with an exposed tendon using 3D‐AMHAT (iv). Reproduced with permission.^[^ ^169b^ ^]^ Copyright 2023, MDPI, Basel, Switzerland.

Classifying the results of different animal models showed that 3D printing‐based hydrogel dressings had good biocompatibility and promoted the healing of mouse wounds in ≈2 weeks by exerting multiple functions (**Table** **5**). In addition to evaluating in vitro cell cultures and in vivo animal studies, the clinical implementation of the technology is necessary because clinical studies can reveal important information, such as treatment effectiveness, cost, safety, and treatment risks. The purpose of conducting clinical research is to assess the potential of 3D hydrogel dressings in wound management and control, thereby transferring this technology from laboratory research to clinical application and promoting the continuous advancement of emerging strategies for wound treatment.^[^ ^32^ ^]^ We attempted to summarize the preliminary trial results of the clinical applications of 3D‐printed hydrogel dressings based on autologous adipose tissue in recent years (**Figure** **15** and Table 5).^[^ ^169^ ^]^ Armstrong et al.^[^ ^169a^ ^]^ used 3D printed autologous minimally invasive processed homologous adipose tissue (AMHAT) to conduct a single‐arm preliminary study of 10 patients with a history of chronic DFU as shown in Figure 15a. After 12 weeks of follow‐up, the patient's average wound area reduction rate was 78.3%, and ≈60% of the wounds healed completely without adverse events reported. On this basis, Bajuri et al.^[^ ^169b^ ^]^ combined AMHAT and 3D printed FIB gel scaffolds to conduct a clinical trial of wound healing in 10 patients with DFU (Figure 15b). 70% of patients showed complete wound healing within 12 weeks (at 2, 4, 5, 10, and 12 weeks, respectively) with no adverse events observed, demonstrating the ability of 3D printed AMHAT/FIB gel scaffolds to promote wound healing through high‐quality skin reconstruction. Kim et al.^[^ ^169c^ ^]^ constructed a 3D‐printed scaffold with a minimally manipulated autologous extracellular matrix (MA‐ECM) for diabetic foot wound treatment and achieved excellent wound closure within 4 weeks. These latest clinical trials demonstrate the clinical application potential of 3D printing‐based hydrogel dressings in treating wounds.

**Table 5:** Therapeutic effects of 3D printing‐based hydrogel dressings in various models.

Model		Bioinks	Primary functions	Treatment effects			References
				Wound healing rate (%)	Infection control	Recovery time (d)	
Animal model healing analysis	Full‐thickness skin defect	CS‐Alg	Hemostasis, antibacterial	97.6 ± 1.1	99.7 ± 0.5%	10	[116]
		HAMA‐PBA/GelMA	Self‐healing, antibacterial	93.42	D_zone_/D_hydrogel_: ≈1.5	14	[133]
		Alg‐Gel/PCL/PDA	NIR‐responsive drug release	>90	–	14	[137]
		Spidroin/PFKU/avGel	NIR‐responsive drug release	>95	–	9	[138]
	Full‐thickness skin defect diabetic wound	SA/polymerized PAM	Anti‐inflammatory, antioxidant	Close to 100	Close to 100%	21	[60]
		Concentrated CMC	Self‐healing, drug‐loading, angiogenesis	88.6 ± 4.7	–	14	[87c]
		GelMA	Anti‐inflammatory, antioxidant	Close to 100	–	21	[124]
	Infected full‐thickness skin defect	PCLMA/HAMA/heparin‐SH	Infection‐responsive drug release	>90	>95%	14	[58]
		Alg‐DA/Gelatin	Antibacterial, angiogenesis	>95	>95%	14	[164]
		GelMA‐DA/rGO@PDA	NIR‐response, antibacterial, hemostasis	>95	>99.3%	14	[171]
	Infected full‐thickness skin defect diabetic wound	Dextran/MoS_2_	Antibacterial, antioxidant, self‐healing	96.8±1.04	Close to 100%	15	[129]
	Partial thickness burn wound	CS/methacrylate	Drug‐loading, antibacterial	>90	≈4 cm	21	[127]
	Infected burn wound	CMC/O‐AlgCat	Antibacterial, pH‐response	Close to 100	100%	18	[132]
Clinical model healing analysis	Chronic diabetic foot‐DFU	AMHAT	Anti‐inflammatory, epithelization	100 (60% of patients)	–	49.1 (29.9‐68.3)	[169a]
		AMHAT/FIB gel scaffold		100 (70% of patients)	–	49 (14‐84)	[169b]
		MA‐ECM	Regenerative, reparative	100	–	28	[169c]

Although much research has been performed to develop new multi‐functional 3D printing‐based hydrogel dressings, only a few products have been clinically tested. This is because 3D‐printed products need to go through a strict regulatory process to ensure their safety, effectiveness, and stability. However, sound regulatory boundaries for 3D printing‐based hydrogel dressings are lacking. The International Organization for Standardization‐261 and the American Society for Testing and Materials‐F47 are working to develop common standards for additive manufacturing frameworks, including guidelines, safety, definitions, and concepts, and applicable materials, including material types, processes, and application details, to continuously improve global standards and the quality of additive‐manufactured products.^[^ ^106^ ^]^ Legislation or standards specifically for the application of 3D‐printed wound dressings need to adapt to the advanced nature and complexity of the products. 3D printing‐based hydrogel dressings involving different types of cells (stem cells, fat cells, nerve cells, etc.) will inevitably face more stringent ethical reviews, which will be greatly different from the standard requirements of traditional therapeutic drugs.^[^ ^170^ ^]^ Thus, more scientific supervision and longer‐term research are needed to determine the clinical efficacy and standards of 3D printing‐based hydrogel dressings for clinical translational applications.

## 6 Conclusion and Future Perspectives

This review comprehensively summarized the recent progress in multi‐functional 3D printing‐based hydrogel dressings customized by a variety of 3D printing technologies for wound healing. Skin wound healing stages and assessment indicators were introduced, and the representative 3D printing technology, types of biological ink, and the corresponding design methods were discussed. Finally, the recent progress in the application of multi‐functional 3D printing‐based hydrogel dressings for wound healing was reviewed. 3D printing‐based hydrogel dressings have promising application prospects to promote tissue regeneration and wound healing. However, problems remain to be addressed to fully explore the future potential of 3D printing‐based hydrogel dressings.

One of the main challenges of 3D printing is choosing the appropriate type of biological ink. Natural ink has good biocompatibility but poor mechanical properties, whereas synthetic polymer ink generally has better physical, chemical, and mechanical properties, but its biodegradability needs to be improved. In addition, the structural and conformational changes of human skin (epidermis and dermis) are complex, and each layer of skin varies in composition and function among people of different ages or races. Therefore, designing and printing hydrogel‐based dressings suitable for each patient's skin tissue with good biocompatibility, multi‐functions, and dynamic characteristics is challenging. A deeper understanding of skin biology, the discovery of new biomaterial inks, and the development of cutting‐edge printing technologies, such as in situ bioprinting or dynamic 4D bioprinting that introduces time into the fourth dimension, are likely to provide solutions to this limitation.

Second, 3D printing‐based hydrogel dressing sensor systems, which can perform the real‐time monitoring of the corresponding indexes in each stage of the continuous, dynamic wound healing process, need further technical optimization. These features include the biocompatibility of the sensing system, the adhesion and deformation adaptability to wounds of different shapes and sizes, the design of the electrode structure, and the external transmission of feedback signals. The development of AI‐assisted technology should be focused on, especially the organic combination of ML, 3D printing hydrogel and wound healing, to ensure clear and precise real‐time monitoring and the prediction of wound states and ultimately achieve intelligent and accurate treatment.

Finally, many 3D printing‐based hydrogel dressings with excellent performance have been reportedfor application to mouse wounds, but great biological differences exist between the human body and the commonly used rodent (mouse) models. Moreover, ethical and operating cost issues, and deficiencies in medical supervision in clinical trials of 3D printing‐based hydrogel dressings exist.^[^ ^170^ ^]^ Thus, in vivo models that are more similar to humans, such as pigs and other large mammals, should be used in experimental processes. Establishing quality evaluation standards, supervision, and management systems for 3D printing‐based hydrogel dressings while promoting collaborative innovation between the government, scientific research institutes, and various medical‐related units is also needed to fully tap the market potential of 3D printing‐based hydrogel dressings and further promote the clinical translation.

## Conflict of Interest

The authors declare no conflict of interest.

## References

[1] a) M. Chekini , E. Krivoshapkina , L. Shkodenko , E. Koshel , M. Shestovskaya , M. Dukhinova , S. Kheiri , N. Khuu , E. Kumacheva , Chem. Mater. 2020, 32, 10066;

[2] a) S. Masri , M. Zawani , I. Zulkiflee , A. Salleh , N. I. M. Fadilah , M. Maarof , A. P. Y. Wen , F. Duman , Y. Tabata , I. A. Aziz , Int. J. Mol. Sci. 2022, 23, 476; 35008902 10.3390/ijms23010476 PMC8745539

[3] E. Eriksson , P. Y. Liu , G. S. Schultz , M. M. Martins‐Green , R. Tanaka , D. Weir , L. J. Gould , D. G. Armstrong , G. W. Gibbons , R. Wolcott , Wound Repair Regener. 2022, 30, 156. 10.1111/wrr.12994 PMC9305950 35130362

[4] K. Järbrink , G. Ni , H. Sönnergren , A. Schmidtchen , C. Pang , R. Bajpai , J. Car , Syst. Rev. 2017, 6, 15. 28118847 10.1186/s13643-016-0400-8 PMC5259833

[5] a) A. Markiewicz‐Gospodarek , M. Kozioł , M. Tobiasz , J. Baj , E. Radzikowska‐Büchner , A. Przekora , Int. J. Environ. Res. Public Health 2022, 19, 1338; 35162360 10.3390/ijerph19031338 PMC8834952

[6] G. D. Winter , J. Wound Care 1995, 193, 366. 7553187

[7] a) J. Zhou , D. Yao , Z. Qian , S. Hou , L. Li , A. T. A. Jenkins , Y. Fan , Biomaterials 2018, 161, 11; 29421548 10.1016/j.biomaterials.2018.01.024

[8] a) A. GhavamiNejad , N. Ashammakhi , X. Y. Wu , A. Khademhosseini , Small 2020, 16, 2002931; 10.1002/smll.202002931 PMC7754762 32734720

[9] a) A. Ghaee , S. Bagheri‐Khoulenjani , H. A. Afshar , H. Bogheiri , Composites, Part B 2019, 177, 107339;

[10] a) Y. Li , H. Y. Yang , D. S. Lee , J. Controlled Release 2021, 330, 151; 10.1016/j.jconrel.2020.12.008 33309972

[11] a) F. Zhou , Y. Hong , R. Liang , X. Zhang , Y. Liao , D. Jiang , J. Zhang , Z. Sheng , C. Xie , Z. Peng , Biomaterials 2020, 258, 120287; 32847683 10.1016/j.biomaterials.2020.120287

[12] a) Y. Wang , X. Yuan , B. Yao , S. Zhu , P. Zhu , S. Huang , Bioact. Mater. 2022, 17, 178; 35386443 10.1016/j.bioactmat.2022.01.024 PMC8965032

[13] a) B. Zheng , Z. Qiu , J. Xu , X. Zeng , K. Liu , L. Chen , J Mater. Chem. B 2023, 11, 8411; 37463000 10.1039/d3tb01189e

[14] a) S. Vijayavenkataraman , W. Lu , J. Fuh , Biofabrication 2016, 8, 032001; 27606434 10.1088/1758-5090/8/3/032001

[15] S. Tavakoli , A. S. Klar , Biomolecules 2020, 10, 1169. 32796593 10.3390/biom10081169 PMC7464761

[16] a) R. F. Pereira , A. Sousa , C. C. Barrias , A. Bayat , P. L. Granja , P. J. Bártolo , Biomanuf. Rev. 2017, 2, 1;

[17] B. K. Sun , Z. Siprashvili , P. A. Khavari , Science 2014, 346, 941. 25414301 10.1126/science.1253836

[18] H. N. Wilkinson , M. J. Hardman , Open Biol. 2020, 10, 200223. 32993416 10.1098/rsob.200223 PMC7536089

[19] a) Y. Liang , X. Zhao , T. Hu , B. Chen , Z. Yin , P. X. Ma , B. Guo , Small 2019, 15, 1900046; 10.1002/smll.201900046 30786150

[20] a) S. Y. Wang , H. Kim , G. Kwak , H. Y. Yoon , S. D. Jo , J. E. Lee , D. Cho , I. C. Kwon , S. H. Kim , Adv. Sci. 2018, 5, 1800852; 10.1002/advs.201800852 PMC6247053 30479928

[21] D. S. Masson‐Meyers , T. A. Andrade , G. F. Caetano , F. R. Guimaraes , M. N. Leite , S. N. Leite , M. A. C. Frade , Int. J. Exp. Pathol. 2020, 101, 21. 32227524 10.1111/iep.12346 PMC7306904

[22] a) S. Wang , H. Yang , Z. Tang , G. Long , W. Huang , Stem Cells Int. 2016, 2016, 3269267; 26880953 10.1155/2016/3269267 PMC4736208

[23] a) H. Xu , S. Huang , J. Wang , Y. Lan , L. Feng , M. Zhu , Y. Xiao , B. Cheng , W. Xue , R. Guo , Int. J. Biol. Macromol. 2019, 137, 433; 31271797 10.1016/j.ijbiomac.2019.06.246

[24] S. H. Tan , D. A. C. Chua , J. R. J. Tang , C. Bonnard , D. Leavesley , K. Liang , Acta Biomater. 2022, 153, 13. 36191774 10.1016/j.actbio.2022.09.068

[25] D. K. Chang , M. R. Louis , A. Gimenez , E. M. Reece , Semin. Plast. Surg. 2019, 33, 185. 31384234 10.1055/s-0039-1693401 PMC6680073

[26] Z. H. Li , G. F. Wu , H. Q. Song , K. Huang , B. Wu , X. L. Xu , L. X. Zhu , Med. Sci. Monit. 2022, 28, 936186. 10.12659/MSM.936186 PMC9186093 35661102

[27] a) A. Ali Zahid , A. Chakraborty , Y. Shamiya , S. P. Ravi , A. Paul , Mater. Horiz. 2022, 9, 1850; 35485266 10.1039/d2mh00115b

[28] H. S. Kim , X. Sun , J. H. Lee , H. W. Kim , X. Fu , K. W. Leong , Adv. Drug Delivery Rev. 2019, 146, 209. 10.1016/j.addr.2018.12.014 30605737

[29] a) J. Nie , B. Pei , Z. Wang , Q. Hu , Carbohydr. Polym. 2019, 205, 225; 30446099 10.1016/j.carbpol.2018.10.033

[30] a) M. Hamidi , A. Azadi , P. Rafiei , Adv. Drug Delivery Rev. 2008, 60, 1638; 10.1016/j.addr.2008.08.002 18840488

[31] a) Z. Shi , X. Gao , M. W. Ullah , S. Li , Q. Wang , G. Yang , Biomaterials 2016, 111, 40; 27721086 10.1016/j.biomaterials.2016.09.020

[32] Z. Wang , X. Liang , G. Wang , X. Wang , Y. Chen , Adv. Mater. 2023, 2304738, 10.1002/adma.202304738. 37566537

[33] D. Kim , Y. Wu , Y. K. Oh , J. Controlled Release 2022, 349, 133. 10.1016/j.jconrel.2022.06.047 35787916

[34] A. Gholamipour‐Shirazi , I. T. Norton , T. Mills , Int. J. Biol. Macromol. 2021, 189, 370. 34450141 10.1016/j.ijbiomac.2021.08.127

[35] J. L. Guo , M. T. Longaker , Gels 2022, 9, 19. 36661787 10.3390/gels9010019 PMC9857994

[36] a) G. Guo , Q. Wu , F. Liu , J. Yin , Z. L. Wu , Q. Zheng , J. Qian , 2022, 32, 2108548;

[37] a) R. D. Pedde , B. Mirani , A. Navaei , T. Styan , S. Wong , M. Mehrali , A. Thakur , N. K. Mohtaram , A. Bayati , A. Dolatshahi‐Pirouz , Adv. Mater. 2017, 29, 1606061; 10.1002/adma.201606061 28370405

[38] a) B. C. Gross , J. L. Erkal , S. Y. Lockwood , C. Chen , D. M. Spence , Anal. Chem. 2014, 86, 3240; 24432804 10.1021/ac403397r

[39] a) W. L. Ng , S. Wang , W. Y. Yeong , M. W. Naing , Trends Biotechnol. 2016, 34, 689; 27167724 10.1016/j.tibtech.2016.04.006

[40] J. M. Loh , Y. J. L. Lim , J. T. Tay , H. M. Cheng , H. L. Tey , K. Liang , Bioact. Mater. 2024, 32, 222. 37869723 10.1016/j.bioactmat.2023.09.022 PMC10589728

[41] a) F. P. Melchels , J. Feijen , D. W. Grijpma , Biomaterials 2010, 31, 6121; 20478613 10.1016/j.biomaterials.2010.04.050

[42] a) M. A. Heinrich , W. Liu , A. Jimenez , J. Yang , A. Akpek , X. Liu , Q. Pi , X. Mu , N. Hu , R. M. Schiffelers , Small 2019, 15, 1805510; 10.1002/smll.201805510 PMC6752725 31033203

[43] H. Quan , T. Zhang , H. Xu , S. Luo , J. Nie , X. Zhu , Bioact. Mater. 2020, 5, 110. 32021945 10.1016/j.bioactmat.2019.12.003 PMC6992881

[44] a) J. F. Xing , M. L. Zheng , X. M. Duan , Chem. Soc. Rev. 2015, 44, 5031; 25992492 10.1039/c5cs00278h

[45] a) R. J. Mondschein , A. Kanitkar , C. B. Williams , S. S. Verbridge , T. E. Long , Biomaterials 2017, 140, 170; 28651145 10.1016/j.biomaterials.2017.06.005

[46] a) S. Vijayavenkataraman , W. C. Yan , W. F. Lu , C. H. Wang , J. Y. H. Fuh , Adv. Drug Delivery Rev. 2018, 132, 296; 10.1016/j.addr.2018.07.004 29990578

[47] a) J. Jang , H. J. Park , S. W. Kim , H. Kim , J. Y. Park , S. J. Na , H. J. Kim , M. N. Park , S. H. Choi , S. H. Park , Biomaterials 2017, 112, 264; 27770630 10.1016/j.biomaterials.2016.10.026

[48] a) I. Matai , G. Kaur , A. Seyedsalehi , A. McClinton , C. T. Laurencin , Biomaterials 2020, 226, 119536; 31648135 10.1016/j.biomaterials.2019.119536

[49] a) A. Shapira , N. Noor , M. Asulin , T. Dvir , Appl. Phys. Rev. 2018, 5, 041112;

[50] J. Saroia , W. Yanen , Q. Wei , K. Zhang , T. Lu , B. Zhang , Bio‐Des. Manuf. 2018, 1, 265.

[51] a) M. Singh , H. M. Haverinen , P. Dhagat , G. E. Jabbour , Adv. Mater. 2010, 22, 673; 20217769 10.1002/adma.200901141

[52] a) B. J. De Gans , P. C. Duineveld , U. S. Schubert , Adv. Mater. 2004, 16, 203;

[53] B. Derby , Annu. Rev. Mater. Res. 2010, 40, 395.

[54] C. Dou , V. Perez , J. Qu , A. Tsin , B. Xu , J. Li , ChemBioEng. Rev. 2021, 8, 517.

[55] J. Li , M. Chen , X. Fan , H. Zhou , J. Transl. Med. 2016, 14, 271. 27645770 10.1186/s12967-016-1028-0 PMC5028995

[56] Y. Q. Lu , J. Xu , Y. Su , H. Fang , J. Q. Liu , S. Y. Lv , Y. Y. Cheng , Y. Nie , W. F. Li , B. Pan , K. D. Song , Int. J. Bioprint. 2023, 9, 689.37125261

[57] J. Lai , Y. Liu , G. Lu , P. Yung , X. Wang , R. S. Tuan , Z. A. Li , Bioact. Mater. 2024, 37, 348. 38694766 10.1016/j.bioactmat.2024.03.033 PMC11061618

[58] Y. Liu , C. He , T. Qiao , G. Liu , X. Li , Q. Wan , Z. Zhu , Y. He , Adv. Funct. Mater. 2024, 34, 2314071.

[59] N. Ashammakhi , A. Hasan , O. Kaarela , B. Byambaa , A. Sheikhi , A. K. Gaharwar , A. Khademhosseini , 2019, 8, 1801048. 10.1002/adhm.201801048 30734530

[60] Z. Chen , S. Song , H. Zeng , Z. Ge , B. Liu , Z. Fan , Chem. Eng. J. 2023, 471, 144649.

[61] S. Derakhshanfar , R. Mbeleck , K. Xu , X. Zhang , W. Zhong , M. Xing , Bioact. Mater. 2018, 3, 144. 29744452 10.1016/j.bioactmat.2017.11.008 PMC5935777

[62] a) K. Lin , D. Zhang , M. H. Macedo , W. Cui , B. Sarmento , G. Shen , Adv. Funct. Mater. 2019, 29, 1804943;

[63] a) Z. Bazrafshan , G. K. Stylios , Int. J. Biol. Macromol. 2019, 129, 693; 30769042 10.1016/j.ijbiomac.2019.02.024

[64] a) S. S. Mathew‐Steiner , S. Roy , C. K. Sen , Bioengineering 2021, 8, 63; 34064689 10.3390/bioengineering8050063 PMC8151502

[65] a) H. A. Multhaupt , B. Leitinger , D. Gullberg , J. R. Couchman , Adv. Drug Delivery Rev. 2016, 97, 28; 10.1016/j.addr.2015.10.013 26519775

[66] a) P. S. Gungor‐Ozkerim , I. Inci , Y. S. Zhang , A. Khademhosseini , M. R. Dokmeci , Biomater. Sci. 2018, 6, 915; 29492503 10.1039/c7bm00765e PMC6439477

[67] a) A. Mehrabi , N. Baheiraei , M. Adabi , Z. Amirkhani , Appl. Biochem. Biotechnol. 2020, 190, 931; 31620995 10.1007/s12010-019-03135-6

[68] a) L. Cheng , B. Yao , T. Hu , X. Cui , X. Shu , S. Tang , R. Wang , Y. Wang , Y. Liu , W. Song , Int. J. Biol. Macromol. 2019, 135, 1107; 31173833 10.1016/j.ijbiomac.2019.06.017

[69] a) Y. Zhang , J. Venugopal , Z.‐M. Huang , C. T. Lim , S. Ramakrishna , Polymer 2006, 47, 2911;

[70] a) W. Huang , S. Ling , C. Li , F. G. Omenetto , D. L. Kaplan , Chem. Soc. Rev. 2018, 47, 6486; 29938722 10.1039/c8cs00187a PMC6113080

[71] a) S. Mehrotra , B. A. de Melo , M. Hirano , W. Keung , R. A. Li , B. B. Mandal , S. R. Shin , Adv. Funct. Mater. 2020, 30, 1907436; 33071707 10.1002/adfm.201907436 PMC7566555

[72] a) A. L. Mohamed , A. A. Soliman , E. A. Ali , N. Y. Abou‐Zeid , A. A. Nada , Int. J. Biol. Macromol. 2020, 163, 888; 32659397 10.1016/j.ijbiomac.2020.07.068

[73] S. Xiong , X. Zhang , P. Lu , Y. Wu , Q. Wang , H. Sun , B. C. Heng , V. Bunpetch , S. Zhang , H. Ouyang , Sci. Rep. 2017, 7, 4288. 28655891 10.1038/s41598-017-04149-y PMC5487355

[74] a) G. D. Prestwich , J. Controlled Release 2011, 155, 193; 10.1016/j.jconrel.2011.04.007 PMC3716467 21513749

[75] Y. Wang , G. Chen , H. Zhang , C. Zhao , L. Sun , Y. Zhao , ACS Nano 2021, 15, 5977. 33856205 10.1021/acsnano.0c10724

[76] a) A. Borzacchiello , L. Russo , B. M. Malle , K. Schwach‐Abdellaoui , L. Ambrosio , Biomed Res. Int. 2015, 2015, 871218; 26090451 10.1155/2015/871218 PMC4452290

[77] a) H. Weng , W. Jia , M. Li , Z. Chen , Carbohydr. Polym. 2022, 294, 119767; 35868789 10.1016/j.carbpol.2022.119767

[78] a) C. C. Piras , D. K. Smith , J. Mater. Chem. B 2020, 8, 8171; 32776063 10.1039/d0tb01005g

[79] a) J. M. Silva , J. R. García , R. L. Reis , A. J. García , J. F. Mano , Acta Biomater. 2017, 51, 279; 28126597 10.1016/j.actbio.2017.01.058 PMC5665021

[80] A. Smandri , A. Nordin , N. M. Hwei , K. Y. Chin , I. Abd Aziz , M. B. Fauzi , Polymers (Basel) 2020, 12, 1782. 32784960 10.3390/polym12081782 PMC7463743

[81] F. Pati , J. Jang , D. H. Ha , S. W. Kim , J. W. Rhie , J. H. Shim , D. H. Kim , D. W. Cho , Nat. Commun. 2014, 5, 3935. 24887553 10.1038/ncomms4935 PMC4059935

[82] a) X. Wang , L. Wang , Q. Wu , F. Bao , H. Yang , X. Qiu , J. Chang , ACS Appl. Mater. Interfaces 2019, 11, 1449; 30543278 10.1021/acsami.8b17754

[83] a) J. Xu , H. Fang , S. Zheng , L. Li , Z. Jiao , H. Wang , Y. Nie , T. Liu , K. Song , Int. J. Biol. Macromol. 2021, 187, 840; 34339783 10.1016/j.ijbiomac.2021.07.162

[84] a) P. R. Turner , E. Murray , C. J. McAdam , M. A. McConnell , J. D. Cabral , ACS Appl. Mater. Interfaces 2020, 12, 32328; 32597164 10.1021/acsami.0c07212

[85] a) D. Klemm , F. Kramer , S. Moritz , T. Lindström , M. Ankerfors , D. Gray , A. Dorris , Angew. Chem., Int. Ed. Engl. 2011, 50, 5438; 21598362 10.1002/anie.201001273

[86] T. Carvalho , G. Guedes , F. L. Sousa , C. S. R. Freire , H. A. Santos , Biotechnol. J. 2019, 14, 1900059. 10.1002/biot.201900059 31468684

[87] a) A. P. C. Almeida , J. P. Canejo , S. N. Fernandes , C. Echeverria , P. L. Almeida , M. H. Godinho , Adv. Mater. 2018, 30, 1703655; 10.1002/adma.201703655 29333680

[88] J. Yuan , X. Lei , C. Yi , H. Jiang , F. Liu , G. J. Cheng , Chem. Eng. J. 2022, 430, 132765.

[89] a) S. Xin , D. Chimene , J. E. Garza , A. K. Gaharwar , D. L. Alge , Biomater. Sci. 2019, 7, 1179; 30656307 10.1039/c8bm01286e PMC9179007

[90] F. M. Veronese , G. Pasut , Drug Discovery Today 2005, 10, 1451. 16243265 10.1016/S1359-6446(05)03575-0

[91] a) C. L. Teng , J. Y. Chen , T. L. Chang , S. K. Hsiao , Y. K. Hsieh , K. Villalobos Gorday , Y. L. Cheng , J. Wang , Biofabrication 2020, 12, 035024; 31918413 10.1088/1758-5090/ab69da

[92] a) Y. Yu , Z. Yang , S. Ren , Y. Gao , L. Zheng , J. Mol. Liq. 2020, 299, 112185;

[93] a) X. Qing , G. He , Z. Liu , Y. Yin , W. Cai , L. Fan , P. Fardim , Carbohydr. Polym. 2021, 261, 117875; 33766362 10.1016/j.carbpol.2021.117875

[94] a) X. Zhang , J. Gan , L. Fan , Z. Luo , Y. Zhao , Adv. Mater. 2023, 35, 2210903; 10.1002/adma.202210903 36916986

[95] a) C. Pitt , F. Chasalow , Y. Hibionada , D. Klimas , A. Schindler , J. Appl. Polym. Sci. 1981, 26, 3779;

[96] A. Shafiee , A. S. Cavalcanti , N. T. Saidy , D. Schneidereit , O. Friedrich , A. Ravichandran , E. M. De‐Juan‐Pardo , D. W. Hutmacher , Biomaterials 2021, 268, 120558. 33307369 10.1016/j.biomaterials.2020.120558

[97] R. Rai , M. Tallawi , C. Frati , A. Falco , A. Gervasi , F. Quaini , J. A. Roether , T. Hochburger , D. W. Schubert , L. Seik , Adv. Healthcare Mater. 2015, 4, 2012. 10.1002/adhm.201500154 26270628

[98] a) S. Afewerki , A. Sheikhi , S. Kannan , S. Ahadian , A. Khademhosseini , Bioeng. Transl. Med. 2019, 4, 96; 30680322 10.1002/btm2.10124 PMC6336672

[99] a) J. Visser , F. P. Melchels , J. E. Jeon , E. M. van Bussel , L. S. Kimpton , H. M. Byrne , W. J. Dhert , P. D. Dalton , D. W. Hutmacher , J. Malda , Nat. Commun. 2015, 6, 6933; 25917746 10.1038/ncomms7933

[100] Z. Mu , K. Chen , S. Yuan , Y. Li , Y. Huang , C. Wang , Y. Zhang , W. Liu , W. Luo , P. Liang , X. Li , J. Song , P. Ji , F. Cheng , H. Wang , T. Chen , Adv. Healthcare Mater. 2020, 9, 1901469. 10.1002/adhm.201901469 31994326

[101] C. Branco da Cunha , D. D. Klumpers , W. A. Li , S. T. Koshy , J. C. Weaver , O. Chaudhuri , P. L. Granja , D. J. Mooney , Biomaterials 2014, 35, 8927. 25047628 10.1016/j.biomaterials.2014.06.047

[102] H. Zhang , H. Shen , J. Lan , H. Wu , L. Wang , J. Zhou , Carbohydr. Polym. 2022, 295, 119848. 35988999 10.1016/j.carbpol.2022.119848

[103] Y. Gao , M. Hou , R. Yang , L. Zhang , Z. Xu , Y. Kang , P. Xue , Biomacromolecules 2019, 20, 1334. 30703318 10.1021/acs.biomac.8b01715

[104] N. Ashammakhi , S. Ahadian , F. Zengjie , K. Suthiwanich , F. Lorestani , G. Orive , S. Ostrovidov , A. Khademhosseini , Biotechnol. J. 2018, 13, 1800148. 10.1002/biot.201800148 PMC6433173 30221837

[105] S. Singh , D. Choudhury , F. Yu , V. Mironov , M. W. Naing , Acta Biomater. 2020, 101, 14. 31476384 10.1016/j.actbio.2019.08.045

[106] J. D. Tanfani , J. D. Monpara , S. Jonnalagadda , 2023, 8, 2300411.

[107] a) R. Y. Cheng , G. Eylert , J. M. Gariepy , S. He , H. Ahmad , Y. Gao , S. Priore , N. Hakimi , M. G. Jeschke , A. Günther , Biofabrication 2020, 12, 025002; 32015225 10.1088/1758-5090/ab6413 PMC7042907

[108] H. Dong , B. Hu , W. Zhang , W. Xie , J. Mo , H. Sun , J. Shang , Int. J. Bioprint. 2023, 9, 629. 36636132 10.18063/ijb.v9i1.629 PMC9830995

[109] Q. Yang , B. Gao , F. Xu , Biotechnol. J. 2020, 15, 1900086. 10.1002/biot.201900086 31486199

[110] a) J. J. Kim , D.‐W. Cho , Virtual Phys. Prototyping 2024, 19, 2395470;

[111] L. Faber , A. Yau , Y. Chen , Biofabrication 2023, 16, 012001. 10.1088/1758-5090/acfdd0 PMC10561158 37757814

[112] a) C. Douillet , M. Nicodeme , L. Hermant , V. Bergeron , F. Guillemot , J. C. Fricain , H. Oliveira , M. Garcia , Biofabrication 2022, 14, 025006; 10.1088/1758-5090/ac40ed 34875632

[113] F. Tsegay , M. Elsherif , H. Butt , Polymers (Basel) 2022, 14, 1012. 35267835 10.3390/polym14051012 PMC8912626

[114] a) F. Groeber , M. Holeiter , M. Hampel , S. Hinderer , K. Schenke‐Layland , Adv. Drug Delivery Rev. 2011, 63, 352; 10.1016/j.addr.2011.01.005 21241756

[115] F. Yu , Y. Li , B. Zhao , X. Xie , E.‐N. Mohamed , E.‐H. Hany , Y. Morsi , J. Li , J. Pan , X. Mo , Composites, Part B 2022, 247, 110294.

[116] Z. Cai , Q. Saiding , L. Cheng , L. Zhang , Z. Wang , F. Wang , X. Chen , G. Chen , L. Deng , W. Cui , Bioact. Mater. 2021, 6, 4506. 34027237 10.1016/j.bioactmat.2021.04.039 PMC8134719

[117] L. Xu , Z. Zhang , A. M. Jorgensen , Y. Yang , Q. Jin , G. Zhang , G. Cao , Y. Fu , W. Zhao , J. Ju , R. Hou , Mater. Today Bio 2023, 18, 100550. 10.1016/j.mtbio.2023.100550 PMC9874077 36713800

[118] a) V. Falanga , R. R. Isseroff , A. M. Soulika , M. Romanelli , D. Margolis , S. Kapp , M. Granick , K. Harding , Nat. Rev. Dis. Primers 2022, 8, 50; 35864102 10.1038/s41572-022-00377-3 PMC10352385

[119] M. H. Norahan , S. C. Pedroza‐González , M. G. Sánchez‐Salazar , M. M. Álvarez , G. Trujillo de Santiago , Bioact. Mater. 2023, 24, 197. 36606250 10.1016/j.bioactmat.2022.11.019 PMC9803907

[120] Z. Wu , Y. Hong , ACS Appl. Mater. Interfaces 2019, 11, 33734. 31436081 10.1021/acsami.9b14090

[121] N. Kim , H. Lee , G. Han , M. Kang , S. Park , D. E. Kim , M. Lee , M. J. Kim , Y. Na , S. Oh , S. J. Bang , T. S. Jang , H. E. Kim , J. Park , S. R. Shin , H. D. Jung , Adv. Sci. 2023, 10, 2300816. 10.1002/advs.202300816 PMC10265106 37076933

[122] W. Qiu , Q. Wang , M. Li , N. Li , X. Wang , J. Yu , F. Li , D. Wu , Composites, Part B 2022, 234, 109688.

[123] a) F. Liu , X. Liu , F. Chen , Q. Fu , Prog. Polym. Sci. 2021, 123, 101472;

[124] a) Y. Yang , Y. Liang , J. Chen , X. Duan , B. Guo , Bioact. Mater. 2022, 8, 341; 34541405 10.1016/j.bioactmat.2021.06.014 PMC8427086

[125] F. Xu , W. Sun , W. Ma , W. Wang , D. Kong , Y. K. Chan , Q. Ma , Colloids Surf. B Biointerfaces 2024, 234, 113720. 38157763 10.1016/j.colsurfb.2023.113720

[126] X. Du , L. Wu , H. Yan , Z. Jiang , S. Li , W. Li , Y. Bai , H. Wang , Z. Cheng , D. Kong , L. Wang , M. Zhu , Nat. Commun. 2021, 12, 4733. 34354068 10.1038/s41467-021-24972-2 PMC8342549

[127] L. Cao , Z. Ji , B. Zhang , X. Si , Y. Wang , J. Hao , X. Li , W. Mu , X. Yang , C. Shi , ACS Biomater. Sci. Eng. 2023, 9, 2001. 36930196 10.1021/acsbiomaterials.3c00161

[128] S. S. Biranje , J. Sun , L. Cheng , Y. Cheng , Y. Shi , S. Yu , H. Jiao , M. Zhang , X. Lu , W. Han , Q. Wang , Z. Zhang , J. Liu , ACS Appl. Mater. Interfaces 2022, 14, 3792. 35037458 10.1021/acsami.1c21039

[129] a) D. A. Hickman , C. L. Pawlowski , U. D. S. Sekhon , J. Marks , A. S. Gupta , Adv. Mater. 2018, 30, 1700859; 10.1002/adma.201700859 PMC5831165 29164804

[130] a) R. Hao , Z. Cui , X. Zhang , M. Tian , L. Zhang , F. Rao , J. Xue , Front. Chem. 2022, 9, 839055; 35141209 10.3389/fchem.2021.839055 PMC8818740

[131] a) H. Geckil , F. Xu , X. Zhang , S. Moon , U. Demirci , Nanomedicine (Lond) 2010, 5, 469; 20394538 10.2217/nnm.10.12 PMC2892416

[132] M. Shahriari‐Khalaji , M. Sattar , R. Cao , M. Zhu , Bioact. Mater. 2023, 29, 177. 37520303 10.1016/j.bioactmat.2023.07.008 PMC10384635

[133] L. Zhu , S. Chen , K. Liu , W. Wen , L. Lu , S. Ding , C. Zhou , B. Luo , Chem. Eng. J. 2020, 391, 123524.

[134] W. Cao , S. Peng , Y. Yao , J. Xie , S. Li , C. Tu , C. Gao , Acta Biomater. 2022, 152, 60. 36049625 10.1016/j.actbio.2022.08.048

[135] A. Brites , M. Ferreira , S. Bom , L. Grenho , R. Claudio , P. S. Gomes , M. H. Fernandes , J. Marto , C. Santos , Int. J. Pharm. 2023, 632, 122541. 36566824 10.1016/j.ijpharm.2022.122541

[136] Y. Liang , B. Chitrakar , Z. Liu , X. Ming , D. Xu , H. Mo , C. Shi , X. Zhu , L. Hu , H. Li , Int. J. Bioprint. 2023, 9, 671. 37065671 10.18063/ijb.v9i2.671 PMC10090813

[137] J. H. Teoh , A. Mozhi , V. Sunil , S. M. Tay , J. Fuh , C. H. Wang , 2021, 31, 2105932.

[138] Z. Yang , X. Ren , Y. Liu , Composites, Part B 2021, 227, 109390.

[139] X. Ding , Y. Yu , W. Li , Y. Zhao , Matter 2023, 6, 1000.

[140] a) X. Mao , R. Cheng , H. Zhang , J. Bae , L. Cheng , L. Zhang , L. Deng , W. Cui , Y. Zhang , H. A. Santos , Adv. Sci. 2019, 6, 1801555; 10.1002/advs.201801555 PMC6364594 30775235

[141] M. Isik , E. Karakaya , T. S. Arslan , D. Atila , Y. K. Erdogan , Y. E. Arslan , H. Eskizengin , C. C. Eylem , E. Nemutlu , B. Ercan , M. D'Este , B. O. Okesola , B. Derkus , Adv. Healthcare Mater. 2023, 12, 2203044. 10.1002/adhm.202203044 PMC11468991 37014809

[142] M. Caprioli , I. Roppolo , A. Chiappone , L. Larush , C. F. Pirri , S. Magdassi , Nat. Commun. 2021, 12, 2462. 33911075 10.1038/s41467-021-22802-z PMC8080574

[143] Q. Feng , D. Li , Q. Li , H. Li , Z. Wang , S. Zhu , Z. Lin , X. Cao , H. Dong , ACS Appl. Mater. Interfaces 2022, 14, 15653. 35344348 10.1021/acsami.2c01295

[144] M. Karolina Pierchala , F. B. Kadumudi , M. Mehrali , T. G. Zsurzsan , P. J. Kempen , M. P. Serdeczny , J. Spangenberg , T. L. Andresen , A. Dolatshahi‐Pirouz , ACS Nano 2021, 15, 9531. 33983022 10.1021/acsnano.0c09204

[145] a) R. Dimatteo , N. J. Darling , T. Segura , Adv. Drug Delivery Rev. 2018, 127, 167; 10.1016/j.addr.2018.03.007 PMC6003852 29567395

[146] D. Baykara , E. Pilavci , S. Ulag , O. V. Okoro , L. Nie , A. Shavandi , A. C. Koyuncu , O. B. Ozakpinar , M. Eroglu , O. Gunduz , Eur. Polym. J. 2023, 193, 112105.

[147] C. Liu , Z. Wang , X. Wei , B. Chen , Y. Luo , Acta Biomater. 2021, 131, 314. 34256189 10.1016/j.actbio.2021.07.011

[148] Y. Shao , K. Dong , X. Lu , B. Gao , B. He , ACS Appl. Mater. Interfaces 2022, 14, 56525. 36520168 10.1021/acsami.2c16277

[149] a) D. R. Brigstock , Cells 2021, 10, 1596.34202136

[150] a) C. Motter Catarino , D. C. Schuck , L. Dechiario , P. Karande , Sci. Adv. 2023, 9, adg0297; 10.1126/sciadv.adg0297 PMC10575578 37831765

[151] a) Y. Wang , F. Zhang , B. Yao , L. Hou , Z. Li , W. Song , Y. Kong , Y. Tan , X. Fu , S. Huang , Burns Trauma 2023, 11, tkad032; 37397510 10.1093/burnst/tkad032 PMC10309082

[152] Y. Liu , S. Ji , H. Gao , H. Chen , J. Xiang , S. Cui , C. C. Zouboulis , A. Cai , X. Fu , X. Sun , Signal Transduction Targeted Ther. 2023, 8, 286. 10.1038/s41392-023-01531-3 PMC10400560 37537197

[153] a) M. Zhao , B. Song , J. Pu , T. Wada , B. Reid , G. Tai , F. Wang , A. Guo , P. Walczysko , Y. Gu , Nature 2006, 442, 457; 16871217 10.1038/nature04925

[154] H. Wei , L. Han , R. Yin , T. Yang , Y. Liu , C. Mou , F. Pang , T. Wang , Sens. Actuators, B 2023, 386, 133707.

[155] Y. Zong , B. Zong , R. Zha , Y. Zhang , X. Li , Y. Wang , H. Fang , W. L. Wong , C. Li , Adv. Healthcare Mater. 2023, 12, 2300431. 10.1002/adhm.202300431 37102624

[156] B. Mirani , E. Pagan , B. Currie , M. A. Siddiqui , R. Hosseinzadeh , P. Mostafalu , Y. S. Zhang , A. Ghahary , M. Akbari , Adv. Healthcare Mater. 2017, 6, 1700718. 10.1002/adhm.201700718 PMC5845857 28944601

[157] a) B. Monroy , K. Sanchez , P. Arguello , J. Estupiñán , J. Bacca , C. V. Correa , L. Valencia , J. C. Castillo , O. Mieles , H. Arguello , S. Castillo , F. Rojas‐Morales , Comput. Biol. Med. 2023, 165, 107335; 37633087 10.1016/j.compbiomed.2023.107335

[158] X. T. Zheng , Z. Yang , L. Sutarlie , M. Thangaveloo , Y. Yu , N. Salleh , J. S. Chin , Z. Xiong , D. L. Becker , X. J. Loh , B. C. K. Tee , X. Su , Sci. Adv. 2023, 9, adg6670. 10.1126/sciadv.adg6670 PMC10275586 37327328

[159] L. Wang , M. Zhou , T. Xu , X. Zhang , Chem. Eng. J. 2022, 433, 134625.

[160] F. V. Lavrentev , I. S. Rumyantsev , A. S. Ivanov , V. V. Shilovskikh , O. Y. Orlova , K. G. Nikolaev , D. V. Andreeva , E. V. Skorb , ACS Appl. Mater. Interfaces 2022, 14, 7321. 35080838 10.1021/acsami.1c22470

[161] B. Sargent , M. Jafari , G. Marquez , A. S. Mehta , Y. H. Sun , H. Y. Yang , K. Zhu , R. R. Isseroff , M. Zhao , M. Gomez , Sci. Rep. 2022, 12, 9912. 35705588 10.1038/s41598-022-13925-4 PMC9200721

[162] Z. Zhu , H. S. Park , M. C. McAlpine , Sci. Adv. 2020, 6, aba5575. 10.1126/sciadv.aba5575 PMC7299613 32596461

[163] Y. Hu , Y. Xiong , Y. Zhu , F. Zhou , X. Liu , S. Chen , Z. Li , S. Qi , L. Chen , ACS Appl. Mater. Interfaces 2023, 15, 38230. 37535406 10.1021/acsami.3c04733 PMC10436249

[164] B. Chen , L. Huang , R. Ma , Y. Luo , Biomed. Mater. 2023, 18, 045023. 10.1088/1748-605X/acd977 37236199

[165] J. Yin , J. Zhong , J. Wang , Y. Wang , T. Li , L. Wang , Y. Yang , Z. Zhen , Y. Li , H. Zhang , S. Zhong , Y. Wu , W. Huang , Mater. Today Bio 2022, 16, 100361. 10.1016/j.mtbio.2022.100361 PMC9352972 35937577

[166] S. Sheybanikashani , N. Zandi , D. Hosseini , R. Lotfi , A. Simchi , J. Mater. Chem. B 2024, 12, 784. 38179665 10.1039/d3tb02363j

[167] H. Chen , X. Zhang , L. Shang , Z. Su , Adv. Sci. (Weinh) 2022, 9, 2202173. 35859231 10.1002/advs.202202173 PMC9475551

[168] F. Zhang , Z. Zhang , X. Duan , W. Song , Z. Li , B. Yao , Y. Kong , X. Huang , X. Fu , J. Chang , S. Huang , Bioprinting 2023, 9, 703. 10.18063/ijb.703 PMC10236330 37273992

[169] a) D. G. Armstrong , S. G. Harris , Z. Rasor , C. M. Zelen , J. Kim , M. Swerdlow , A. L. Isaac , Plast. Reconstr. Surg. Glob Open 2022, 10, 4588; 10.1097/GOX.0000000000004588 PMC9616634 36320618

[170] a) D. T. Uchida , M. L. Bruschi , AAPS PharmSciTech 2023, 24, 41; 36698047 10.1208/s12249-023-02503-0 PMC9876655

[171] F. Cheng , L. Xu , X. Zhang , J. He , Y. Huang , H. Li , Int. J. Biol. Macromol. 2024, 260, 129372. 38237818 10.1016/j.ijbiomac.2024.129372

